# Supplementary material for: Catalytic role of in-situ formed C-N species for enhanced Li2CO3 decomposition
Source: Nat Commun. 2024 Apr 22;15:3393. doi: 10.1038/s41467-024-47629-2 (PMC11035688; doi:10.1038/s41467-024-47629-2)
Supplement: Supplementary file 1 — Supplementary Information [file 41467_2024_47629_MOESM1_ESM.pdf]

Supplementary Information for

Catalytic Role of *in-situ* formed C-N Species for Enhanced Li_2CO_3 Decomposition

Fangli Zhang[1,2,3], Wenchao Zhang[1,4*], Jodie A Yuwono[2], David Wexler[3], Yameng Fan[3], Jinshuo Zou[2], Gemeng Liang[2], Liang Sun[2], Zaiping Guo[2*]

Affiliations

[1]School of Metallurgy and Environment, Central South University, Changsha, 410083, China

[2]School of Chemical Engineering, The University of Adelaide, Adelaide, SA, 5005 Australia

[3]Institute for Superconducting & Electronic Materials, University of Wollongong, Faculty of Engineering and Information Science, Wollongong, NSW, 2500 Australia

[4]Chinese National Engineering Research Centre for Control & Treatment of Heavy Metal Pollution, Changsha, 410083, China

*Corresponding author. Email: wenchao.zhang@csu.edu.cn; zaiping.guo@adelaide.edu.au

This PDF file includes:

Supplementary Methods

Supplementary Equations

Supplementary Figures

Supplementary Tables

Supplementary References

Supplementary Methods

Characterization methods. The transmission electron microscope (TEM) data were processed with DigitalMicrograph (Gatan) software. The X-ray absorption spectroscopy data were processed using Igor Pro 8, with the aid of QANT, which was developed at the Australian Synchrotron. For characterizing the cycled cathodes, the cells were disassembled inside an Ar-filled glove box, and the obtained electrodes were thoroughly washed with purified dimethyl sulfoxide (DMSO) to remove the residual lithium salts before these measurements. When doing the X-ray photoelectron spectroscopy (XPS) experiments, ion beam etching with an etching time of 30 seconds was applied before data collection.

Theoretical calculations. The simulation systems used the following ratios for each component:

System	Number of components		
	Li^+	Anion	DMSO
1M $LiNO_3$/DMSO	1	1	14
1M $LiBF_4$/DMSO	1	1	14
1M LiTFSI/DMSO	1	1	14
1M LiFSI/DMSO	1	1	14
1M $LiNO_3$ and LiFSI/DMSO with $LiNO_3$: LiFSI = 1:3	4	NO_3 = 1 FSI = 3	56
1M $LiNO_3$ and LiFSI/DMSO with $LiNO_3$: LiFSI = 1:1	2	NO_3 = 1 FSI = 1	28
1M $LiNO_3$ and LiFSI/DMSO with $LiNO_3$: LiFSI = 3:1	4	NO_3 = 3 FSI = 1	56

The cut-off distance of 1.2 nm was used for the Lennard-Jones potential. The Coulombic potential was measured using Particle Mesh Ewald (PME) summation with a cut-off distance of 1.0 nm and Fourier grid spacing of 0.12. All bonds were constrained with the LINCS algorithm. Periodic boundary conditions were applied in all directions. The molecular dynamics (MD) simulations were started by running initial energy minimization, followed by 500 ps of NVT simulation and 1 ns of NPT simulation with an integration time step of 0.001 ps. All the simulation systems were finally maintained at 298 K using the Nosé-Hoover thermostat for 15 ns to collect simulation data. A time constant of 1 ps was applied for the temperature coupling. The analysis of MD trajectories (i.e., mean square displacement and diffusion coefficient) was performed using standard tools within GROMACS. Several Li ion structures observed from the MD simulations were then investigated using density functional theory (DFT).

Supplementary Equations(1)-(4)

$$2CO_2 + 2e^- \rightarrow C_2O_4^{2-} \quad \boldsymbol{S}(1)$$

$$C_2O_4^{2-} \rightarrow CO_2^{2-} + CO_2 \quad \boldsymbol{S}(2)$$

$$C_2O_4^{2-} + CO_2^{2-} \rightarrow 2CO_3^{2-} + C \quad \boldsymbol{S}(3)$$

$$2Li^+ + CO_3^{2-} \rightarrow Li_2CO_3 \quad \boldsymbol{S}(4)$$

Supplementary Figures

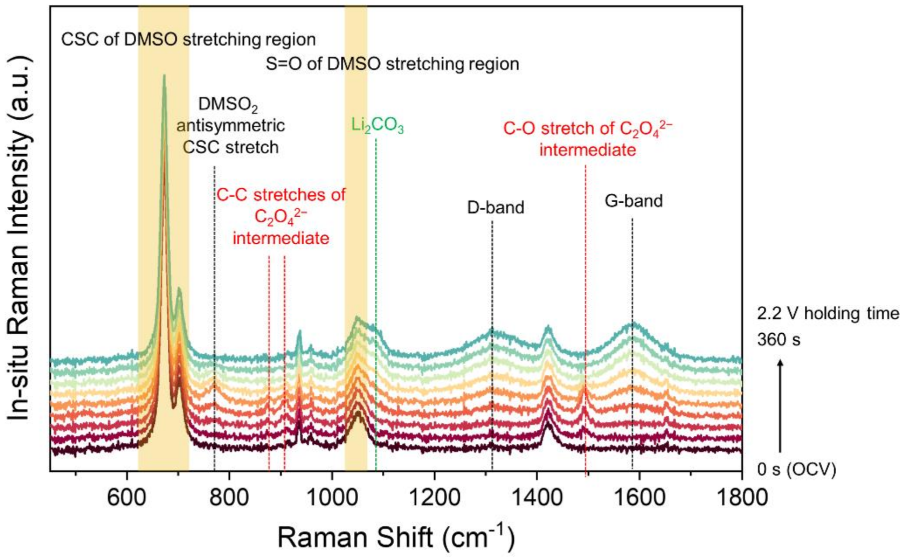

Supplementary Fig. 1. Observation of intermediates during the CO_2 reduction process, via *in-situ* surface-enhanced Raman spectroscopy (SERS).

Note: Although it is hard to directly detect the existence of $*CO_2^{2-}$ due to its instability, especially during battery cycling, $C_2O_4^{2-}$ could be detected as the intermediate at the beginning of discharge. This is revealed in Fig. S1, where the newly-emerged peaks at 876 and 907 cm^{-1} can be assigned to C-C stretching modes, and the pair of peaks at 1492 and 1653 cm^{-1} can be attributed to the C-O stretching modes in $C_2O_4^{2-}$. Subsequently, the $C_2O_4^{2-}$ intermediate gradually disappeared. This was accompanied by the appearance of peaks of 1319 and 1587 cm^{-1} for D and G band, respectively, and the Li_2CO_3 (1089 cm^{-1}), a result consistent with the above reaction pathway of CO_2 reduction (eq S(1)-(4)).

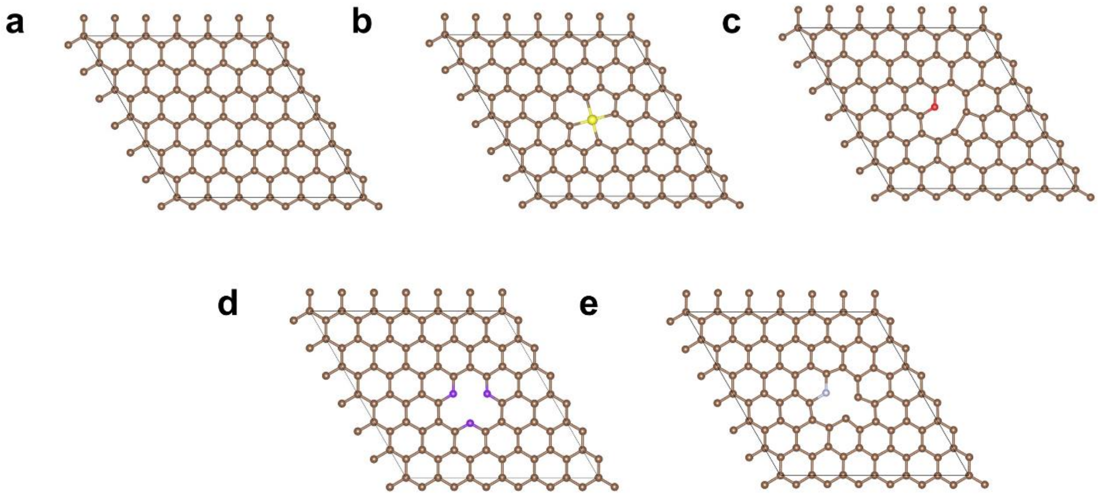

Supplementary Fig. 2. Top view of the atomic structures of (a) graphene, (b) C-S, (c) C-O, (d) C-N, and (e) C-F species. The carbon, oxygen, fluorine, nitrogen, and sulfur atoms are marked as copper, red, silver, purple, and yellow, respectively.

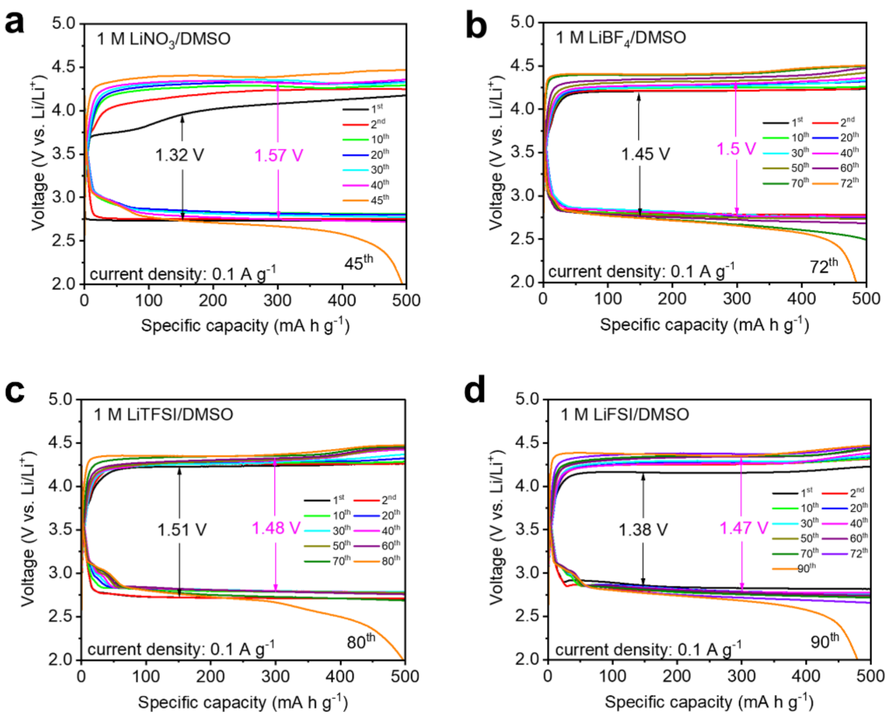

Supplementary Fig. 3. Cycling performance of Li-CO_2 batteries at a current density of 0.1 A g^{-1} with a cut-off specific capacity of 500 mA h g^{-1} (in the cut-off voltage from 2 V to 5 V) in 1 M (a) $LiNO_3$, (b) $LiBF_4$, (c) LiTFSI, and (d) LiFSI electrolytes.

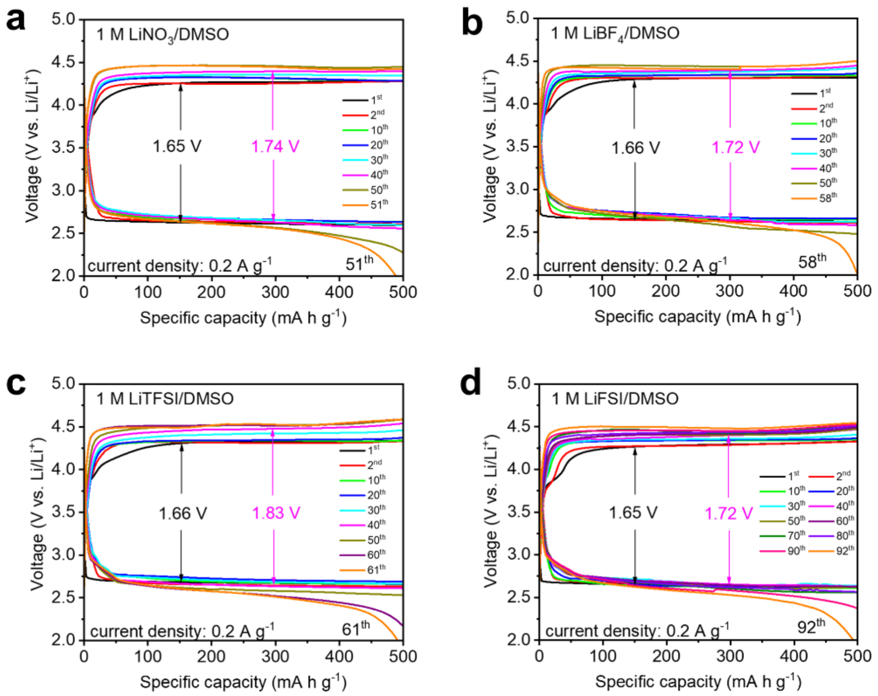

Supplementary Fig. 4. Cycling performance of Li-CO_2 batteries at a current density of 0.2 A g^{-1} with a cut-off specific capacity of 500 mA h g^{-1} (in the cut-off voltage from 2 V to 5 V) in 1 M (a) $LiNO_3$, (b) $LiBF_4$, (c) LiTFSI, and (d) LiFSI electrolytes.

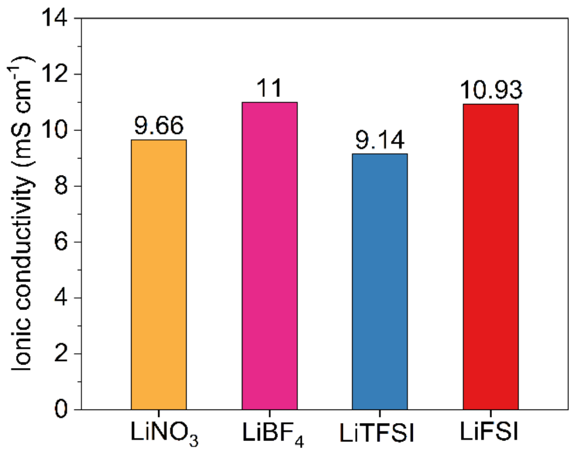

Supplementary Fig. 5. Comparison of ionic conductivities of 1 M single-salt-based electrolytes.

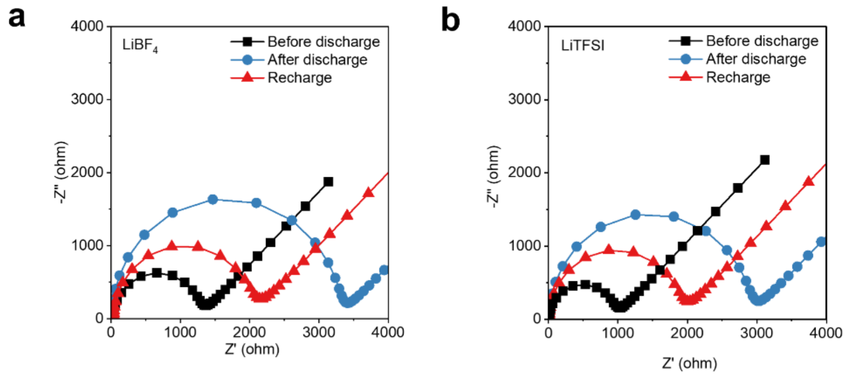

Supplementary Fig. 6. EIS spectra of the (a) $LiBF_4$, and (b) LiTFSI cells before discharge, after the first discharge, and after recharge.

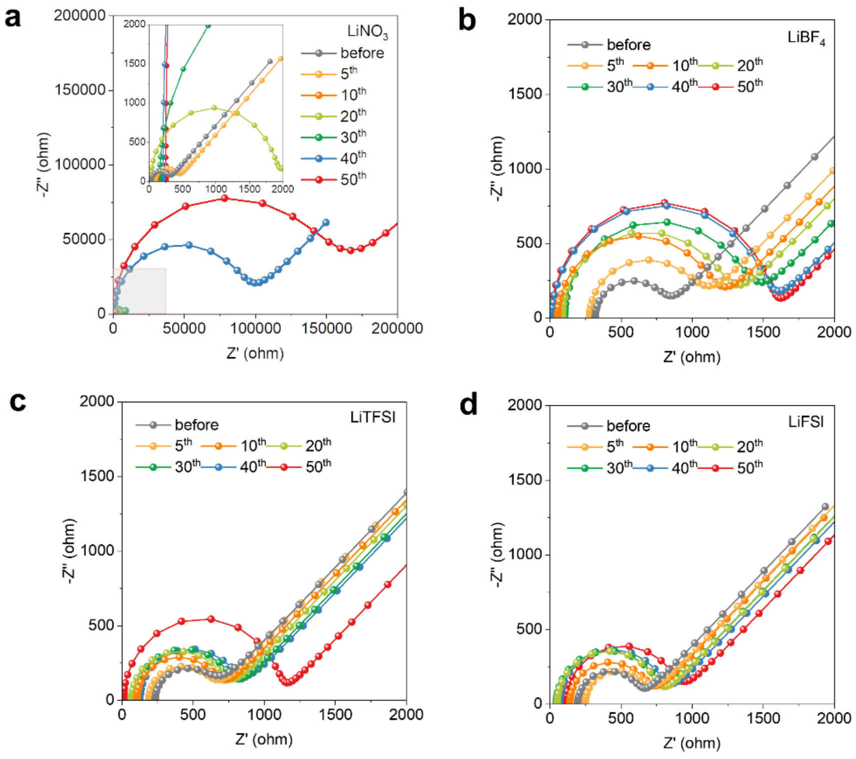

Supplementary Fig. 7. EIS spectra of the Li-CO_2 full-cells in the (a) $LiNO_3$, (b) $LiBF_4$, (c) LiTFSI, and (d) LiFSI electrolytes (fully charged state) with cycling (before cycling, and after 5 cycles, 10 cycles, 20 cycles, 30 cycles, 40 cycles, 50 cycles.). The inset in Fig. S7a shows the enlarged EIS spectra for the cell using the $LiNO_3$ electrolyte.

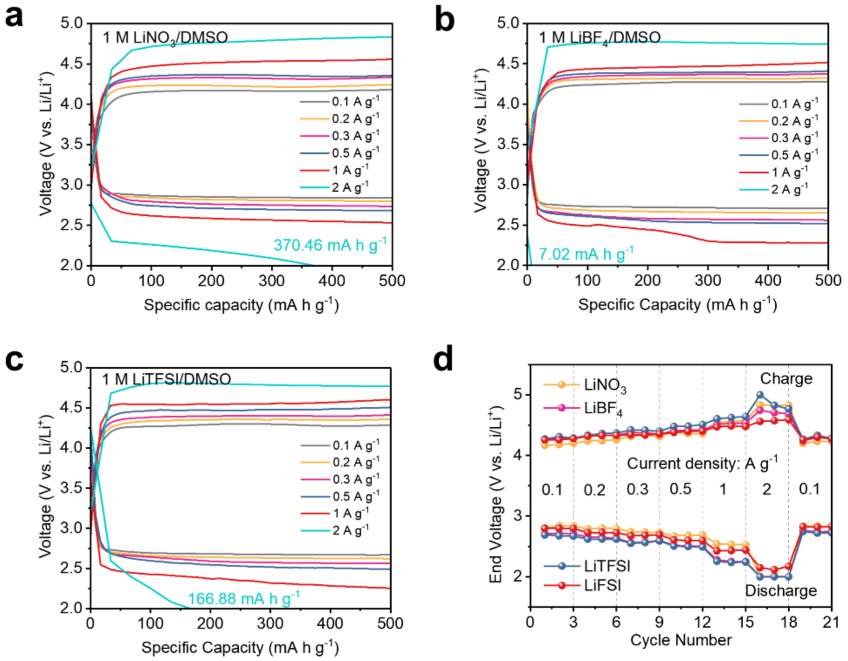

Supplementary Fig. 8. Rate performance of Li-CO_2 batteries at various current densities with a cut-off specific capacity of 500 mA h g^{-1} (in the cut-off voltage from 2 V to 5 V) in (a) $LiNO_3$, (b) $LiBF_4$, and (c) LiTFSI electrolytes. (d) Rate performance with various electrolytes.

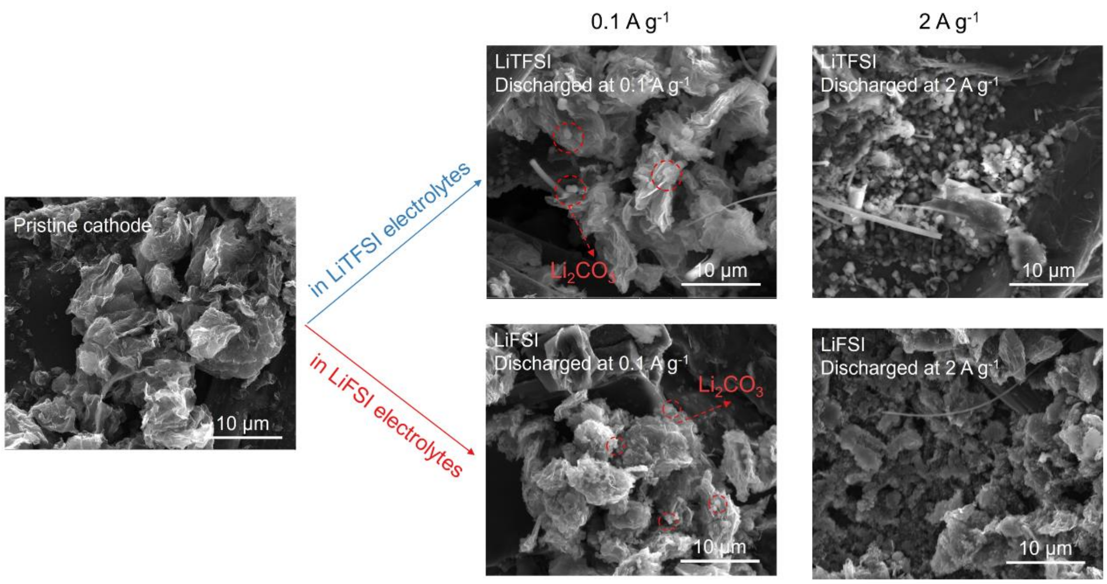

Supplementary Fig. 9. Scanning electron microscope (SEM) images of the pristine cathode and cathodes discharged in the cells using 1 M LiTFSI and LiFSI electrolytes at current densities of 0.1 and 2 A g^{-1}.

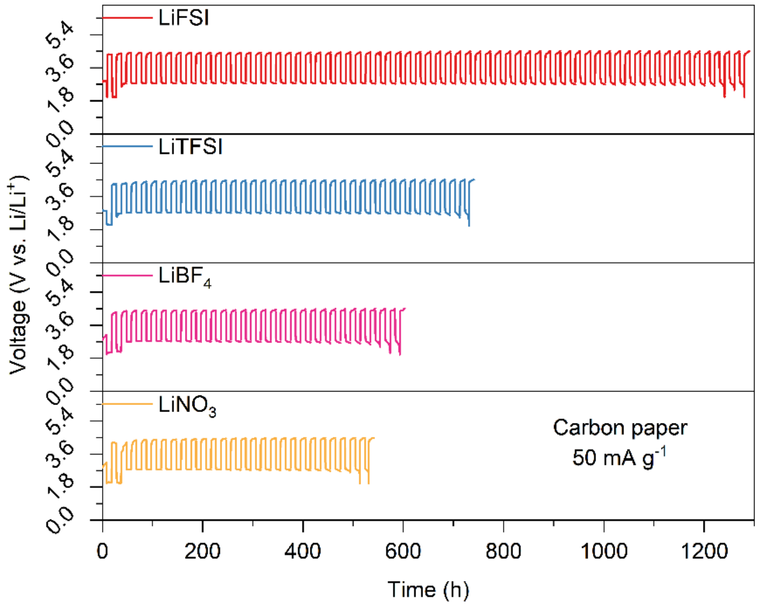

Supplementary Fig. 10. Cycling performance of Li-CO_2 batteries without catalysts at a current density of 50 mA g^{-1} with a cut-off specific capacity of 500 mA h g^{-1} (in the cut-off voltage from 2 V to 5 V) in 1 M $LiNO_3$, $LiBF_4$, LiTFSI, and LiFSI electrolytes.

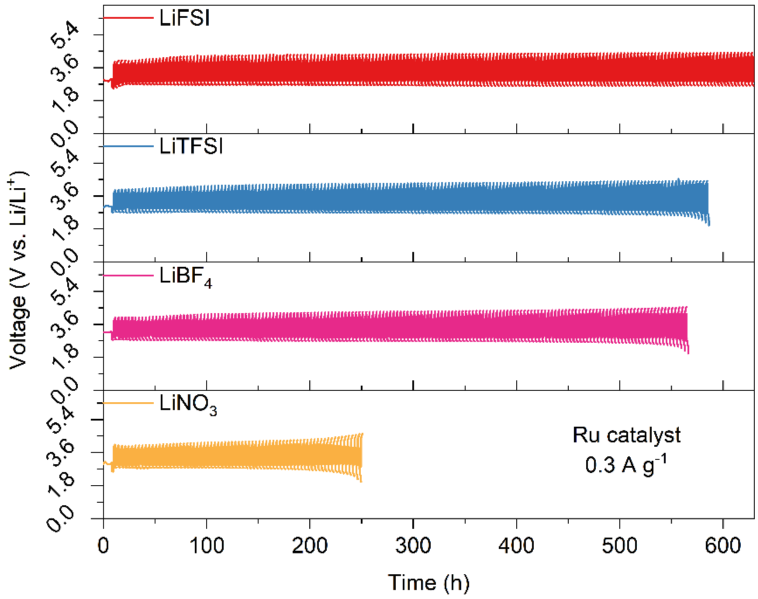

Supplementary Fig. 11. Cycling performance of Li-CO_2 batteries with a ruthenium (Ru) catalyst at a current density of 0.3 A g^{-1} with a cut-off specific capacity of 500 mA h g^{-1} (in the cut-off voltage from 2 V to 5 V) in 1 M $LiNO_3$, $LiBF_4$, LiTFSI, and LiFSI electrolytes.

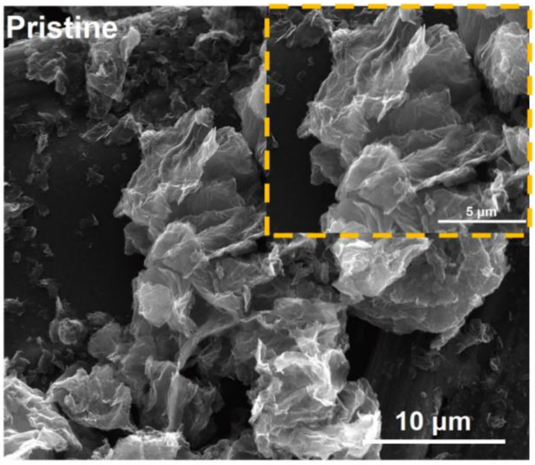

Supplementary Fig. 12. SEM image of the pristine cathode (carbon paper loading with reduced graphene oxide as a catalyst); scale bar, 10 µm. The inset shows an enlarged image; scale bar, 5 µm.

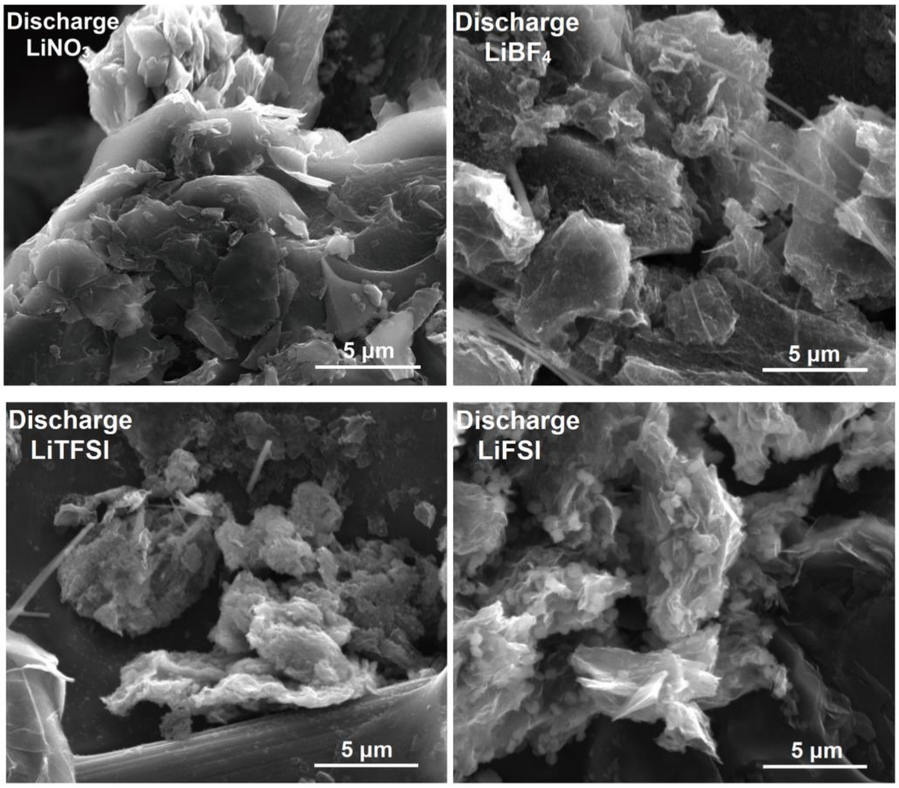

Supplementary Fig. 13. SEM images of the cathodes discharged in the cells using 1 M $LiNO_3$, $LiBF_4$, LiTFSI, and LiFSI electrolytes.

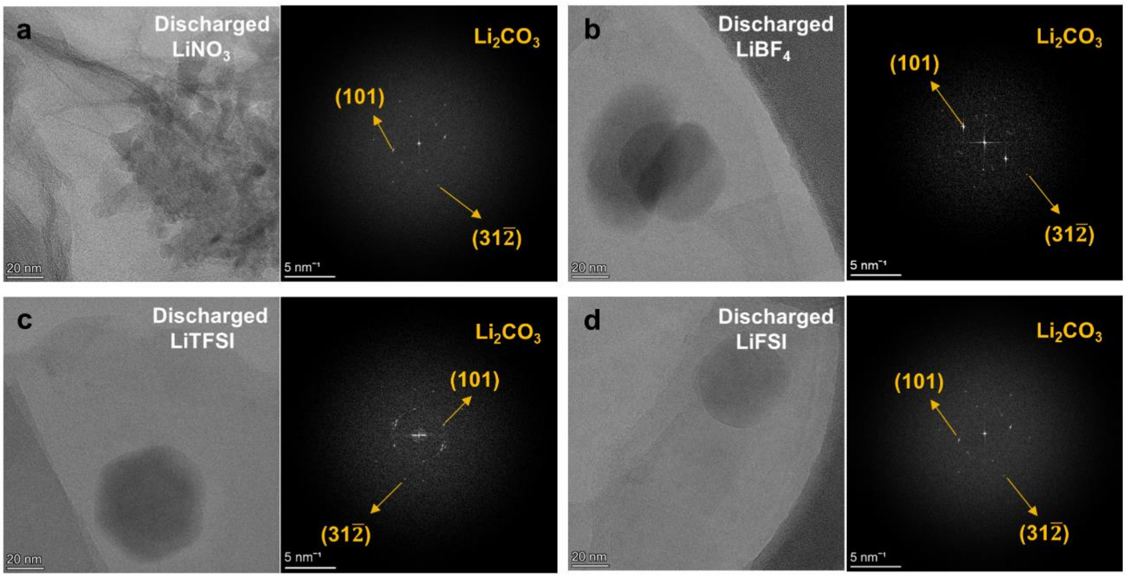

Supplementary Fig. 14. High-resolution transmission electron microscope (HRTEM) images and corresponding inset fast Fourier transform patterns (FFT) of the cathodes discharged in the cells using (a) 1 M $LiNO_3$, (b) $LiBF_4$, (c) LiTFSI, and (d) LiFSI electrolytes.

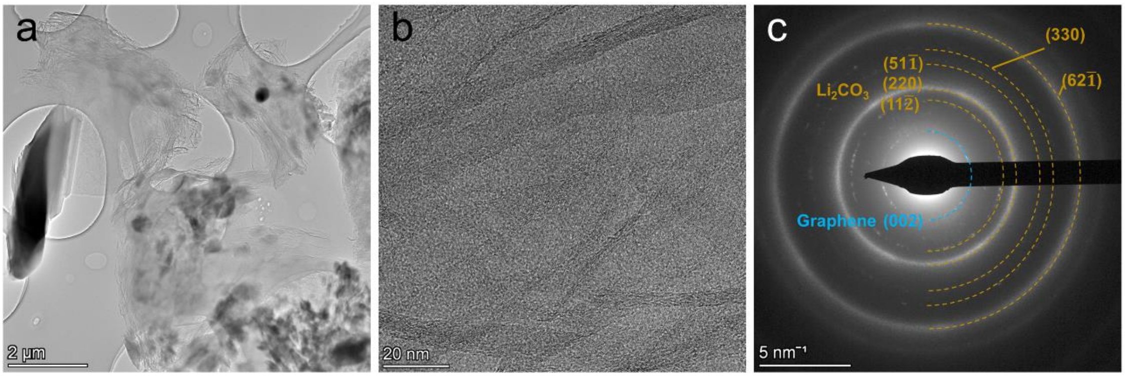

Supplementary Fig. 15. Scanning transmission electron microscopy (STEM) images for the cathodes discharged in the cells using 1 M $LiNO_3$ electrolytes: (a) scale bars, 2 µm; (b) scale bar, 20 nm; and (c) the selected area electron diffraction (SAED) pattern.

Note: The discharge products were observed to be grown on rGO sheets (Fig. S15a). Except for the (002) lattice plane of graphene, other diffraction rings can be confirmed as (112), (220), (51$\bar{1}$), (330), and (62$\bar{1}$) lattice planes of Li_2CO_3 in SAED (Fig. S15c).

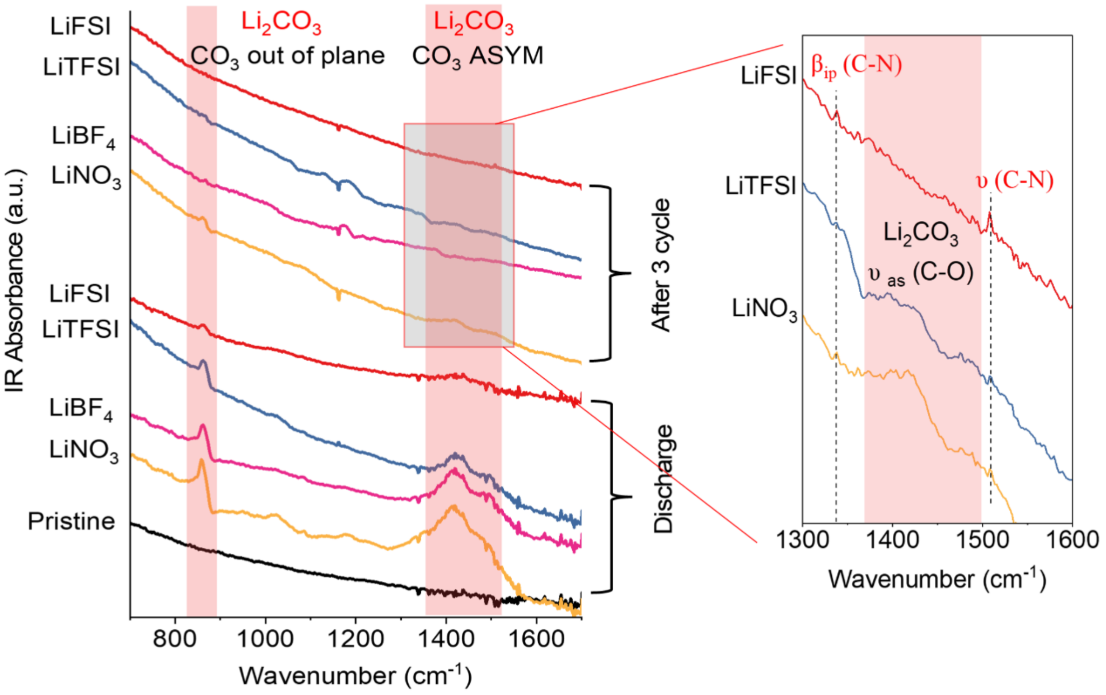

Supplementary Fig. 16. Fourier transform infrared **(**FTIR) spectra of the pristine cathode, the discharged cathodes, and the cathodes after 3 cycles in the cells using 1 M $LiNO_3$, $LiBF_4$, LiTFSI, and LiFSI electrolytes. An enlargement for the cycled cathodes in the range of 1300-1600 cm^{-1} is shown on the right.

Note: After 3 cycles, the peaks at around 1340 and 1510 cm^{-1} (Fig. S16) are assigned to in-plane bending and stretching vibrations of C-N species, respectively,[1-3] confirming the existence of C-N species formed on the surfaces of the cathodes. The FTIR spectra show the highest peak intensities of C-N species on the cathode cycled in the LiFSI-based electrolytes compared to those in the other electrolytes, consistent with the results of no Li_2CO_3 residuals observed in LiFSI-based electrolytes. In contrast, certain amounts of Li_2CO_3 residuals were detected in the $LiNO_3$ and LiTFSI electrolytes with lower intensities of C-N species. These observations identified a positive correlation between C-N species and the reversibility of Li_2CO_3, consistent with the XPS and XANES results in this work.

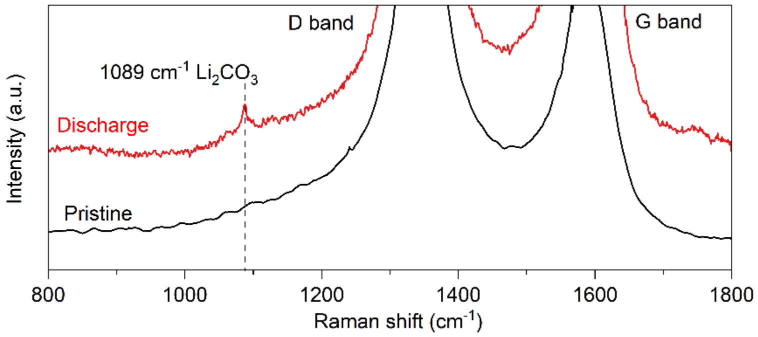

Supplementary Fig. 17. Raman spectra of the cathodes before and after discharge, which were limited to a specific capacity of 500 mA h g^{-1}.

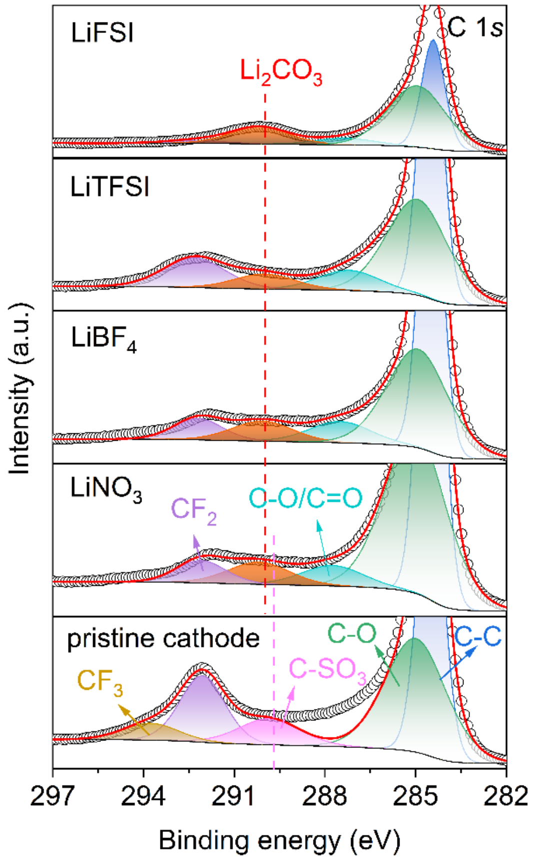

Supplementary Fig. 18. C 1*s* XPS spectra of the pristine cathode (carbon paper loading with reduced graphene oxide) and the cathodes discharged in the cells using 1 M $LiNO_3$, $LiBF_4$, LiTFSI, and LiFSI electrolytes. (The peaks at 284.5 eV and 285 eV can be ascribed to non-oxygenated ring carbon (C-C) and the presence of oxygen in rGO (C-O), respectively; C-O/C=O: 287.7 eV, CF_2: ~293 eV).[4-6]

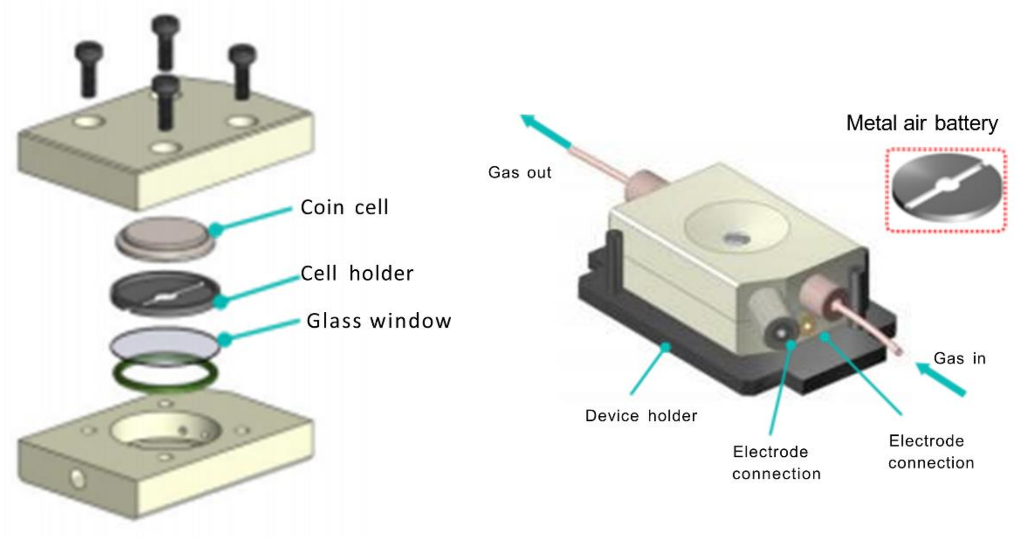

Supplementary Fig. 19. Schematic of *in-situ* Raman cell.

Note: For *in-situ* Raman test, CR2032-type coin cells (a hole on the cathode side) were assembled in an Ar-filled glove box with air electrodes and lithium chip anodes separated by a glass fiber separator (Whatman, diameter: 19 mm). Solutions of 1 M $LiNO_3$/DMSO and 1 M LiFSI/DMSO were used as electrolytes. The as-prepared coin cells were sealed in *in-situ* Raman cell and purged with CO_2 gas during the test process.

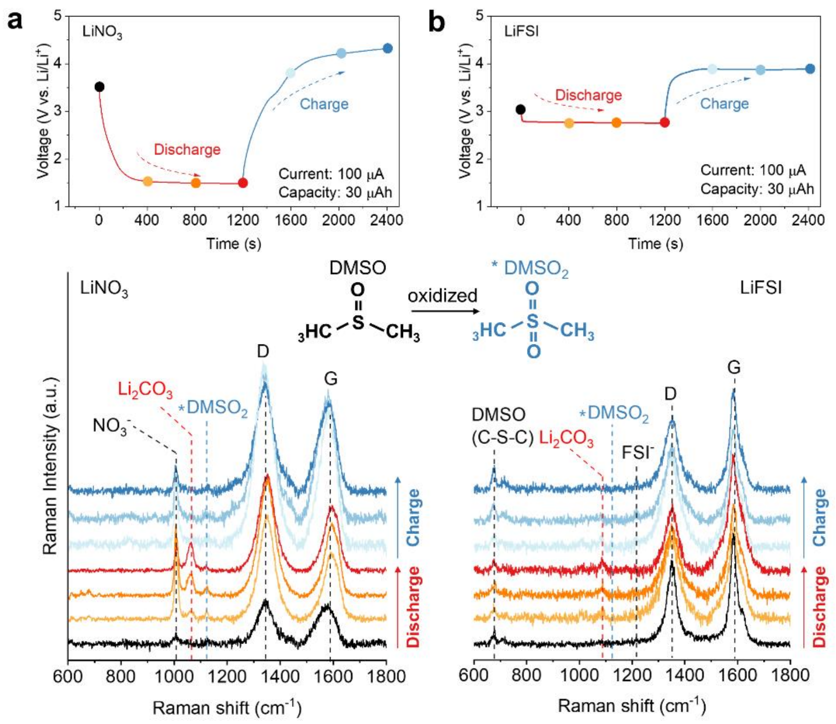

Supplementary Fig. 20. *In-situ* Raman evidence for the Li-CO_2 cells with the formation/decomposition of Li_2CO_3 during discharge/charge. The *in-situ* Raman spectra collected at specific points (marked at voltage profile) during battery running at a constant current of 100 μA with a cut-off capacity of 30 μAh for (a) $LiNO_3$ cells and (b) LiFSI cells. The peaks assigned to dimethyl sulfone ($DMSO_2$) species were observed during cycling. Corresponding voltage profiles are listed above each group of the *in-situ* Raman spectra.

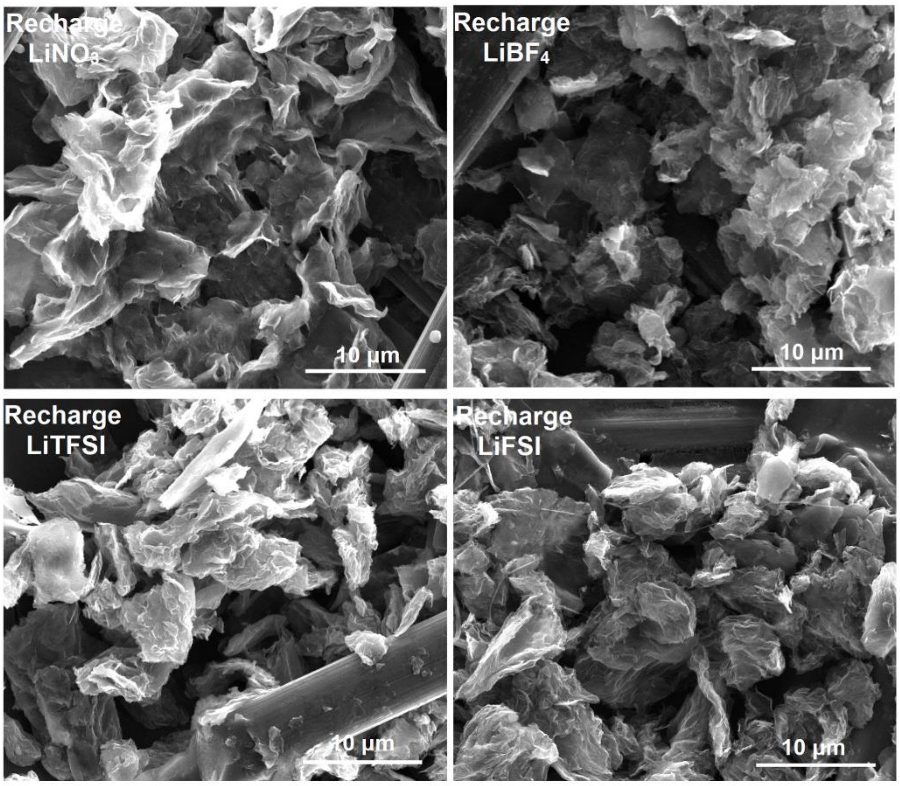

Supplementary Fig. 21. SEM images of the cathodes after 3 cycles in the cells using 1 M $LiNO_3$, $LiBF_4$, LiTFSI, and LiFSI electrolytes.

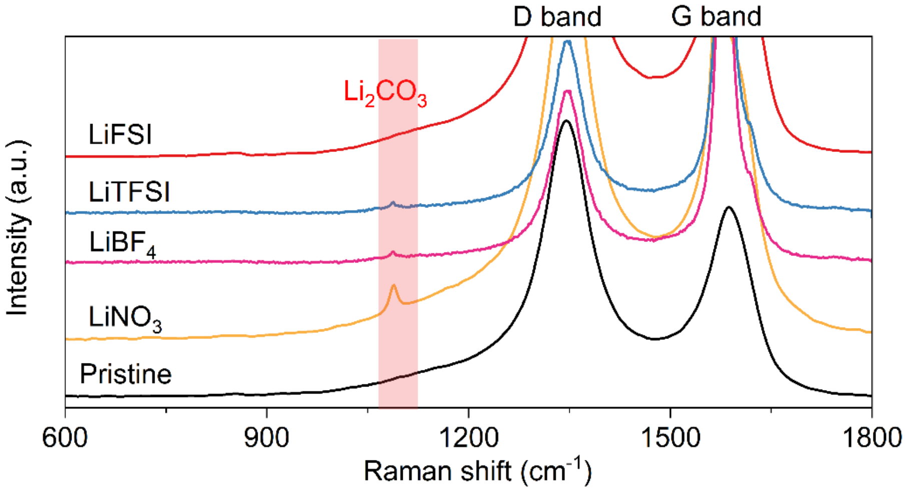

Supplementary Fig. 22. Raman spectra of the cathodes after 3 cycles in the cells using 1 M $LiNO_3$, $LiBF_4$, LiTFSI, and LiFSI electrolytes.

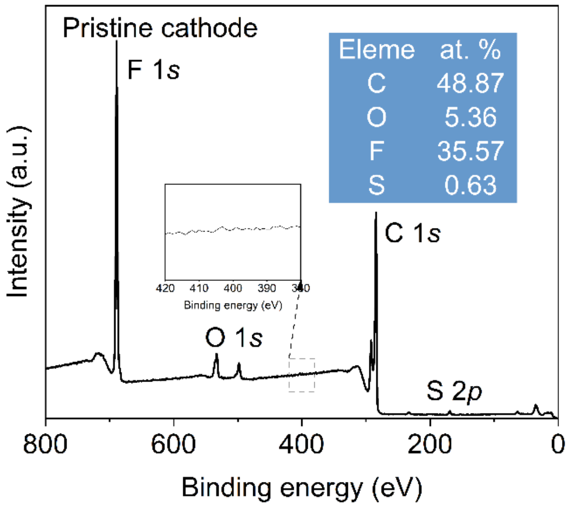

Supplementary Fig. 23. XPS survey spectrum of the pristine cathode, with enlargement in the range of 380-420 eV in the inset.

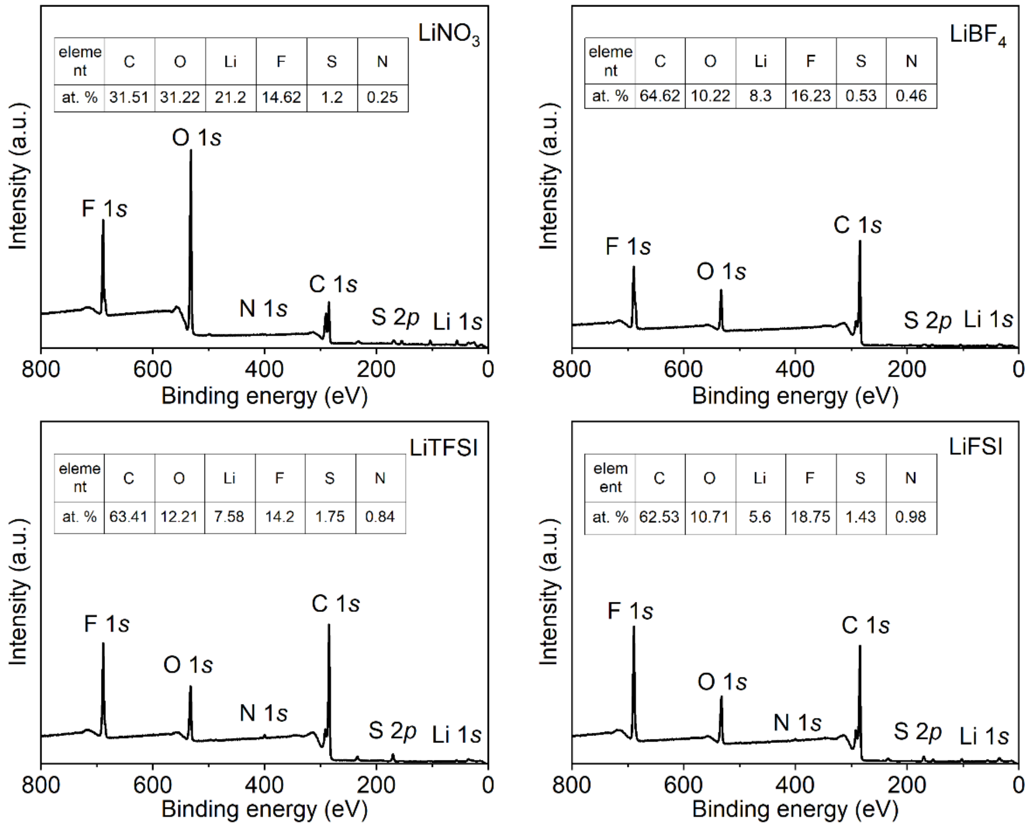

Supplementary Fig. 24. XPS survey spectra of the cathodes after 3 cycles in the cells using 1 M $LiNO_3$, $LiBF_4$, LiTFSI, and LiFSI electrolytes.

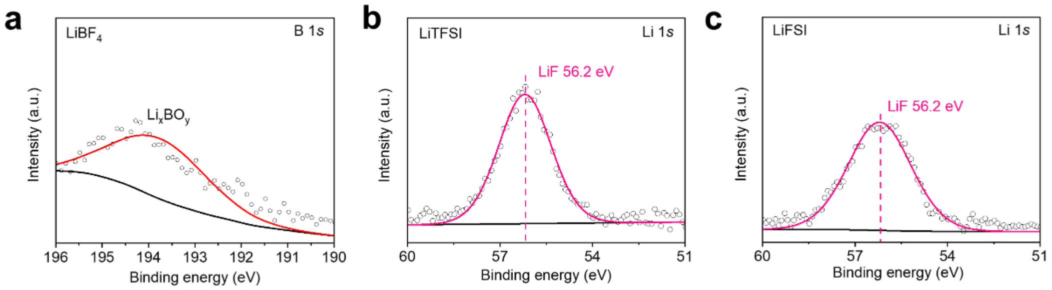

Supplementary Fig. 25. B 1*s* XPS spectrum of the cathode after 3 cycles in the cell using (a) the $LiBF_4$ electrolyte. Li 1*s* XPS spectra of the cathodes after 3 cycles in the cells using (b) LiTFSI and (c) LiFSI electrolytes. The LiF peak is at 56.2 eV.[7]

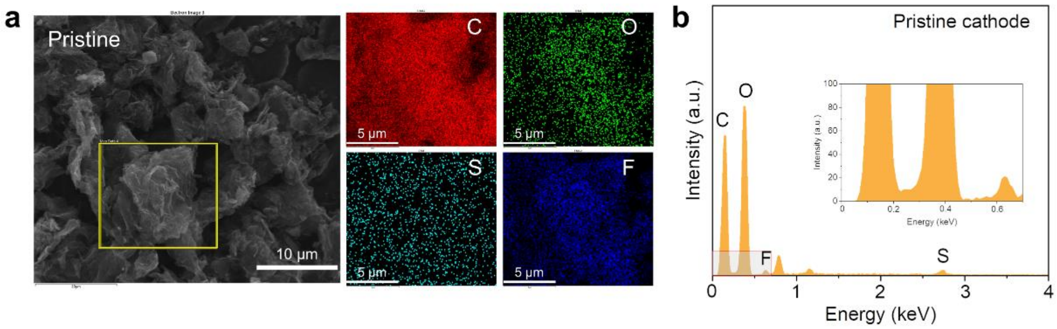

Supplementary Fig. 26. Energy dispersive spectroscopy (EDS) analysis for the pristine cathode (carbon paper loading with reduced graphene oxide): (a) SEM image and EDS spectrum imaging for chemical maps of C, O, S and F; and (b) EDS spectrum with enlargement of the indicated range in the inset.

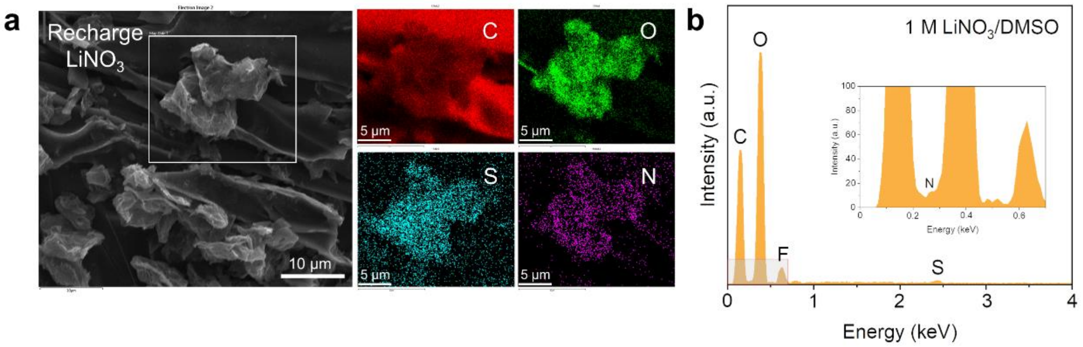

Supplementary Fig. 27. EDS analysis for the cathode after 3 cycles in the $LiNO_3$ electrolyte: (a) SEM image and EDS spectrum imaging for chemical maps of C, O, S and N; and (b) EDS spectrum with enlargement of the indicated range in the inset.

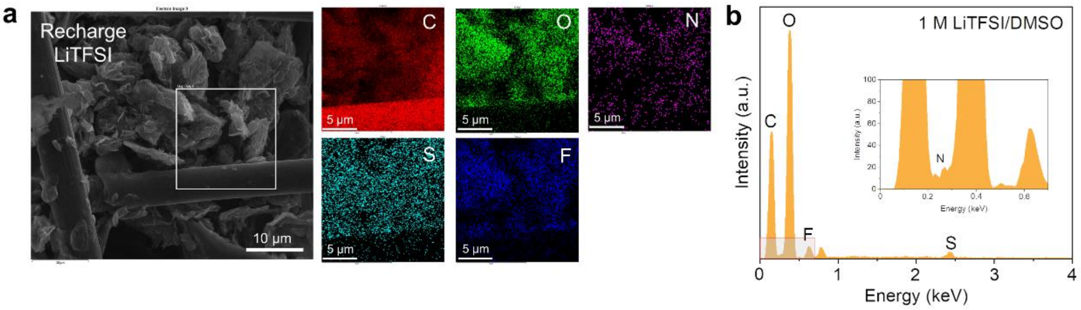

Supplementary Fig. 28. EDS analysis for the cathode after 3 cycles in the LiTFSI electrolyte: (a) SEM image and EDS spectrum imaging for chemical maps of C, O, N, S and F; and (b) EDS spectrum with enlargement of the indicated range in the inset.

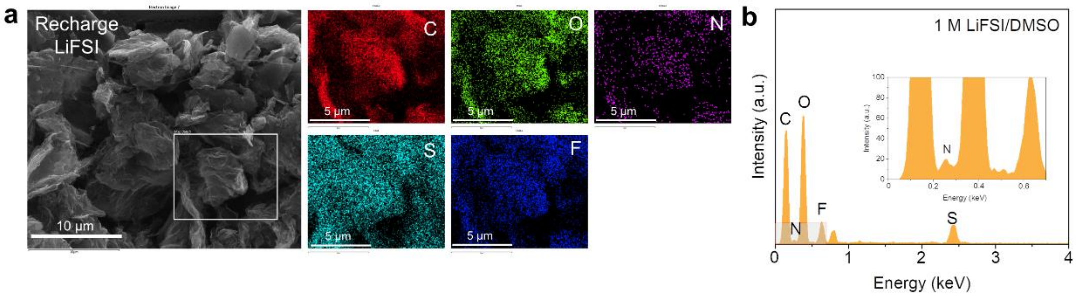

Supplementary Fig. 29. EDS analysis for the cathode after 3 cycles in the LiFSI electrolyte: (a) SEM image and EDS spectrum imaging for chemical maps of C, O, N, S and F; and (b) EDS spectrum with enlargement of the indicated range in the inset.

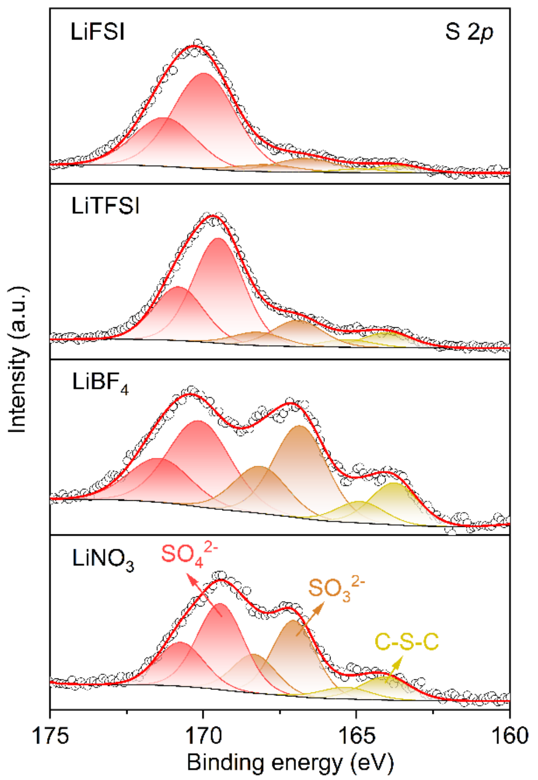

Supplementary Fig. 30. S 2*p* XPS spectra of the cathodes after 3 cycles in the cells using 1M $LiNO_3$, $LiBF_4$, LiTFSI, and LiFSI electrolytes.

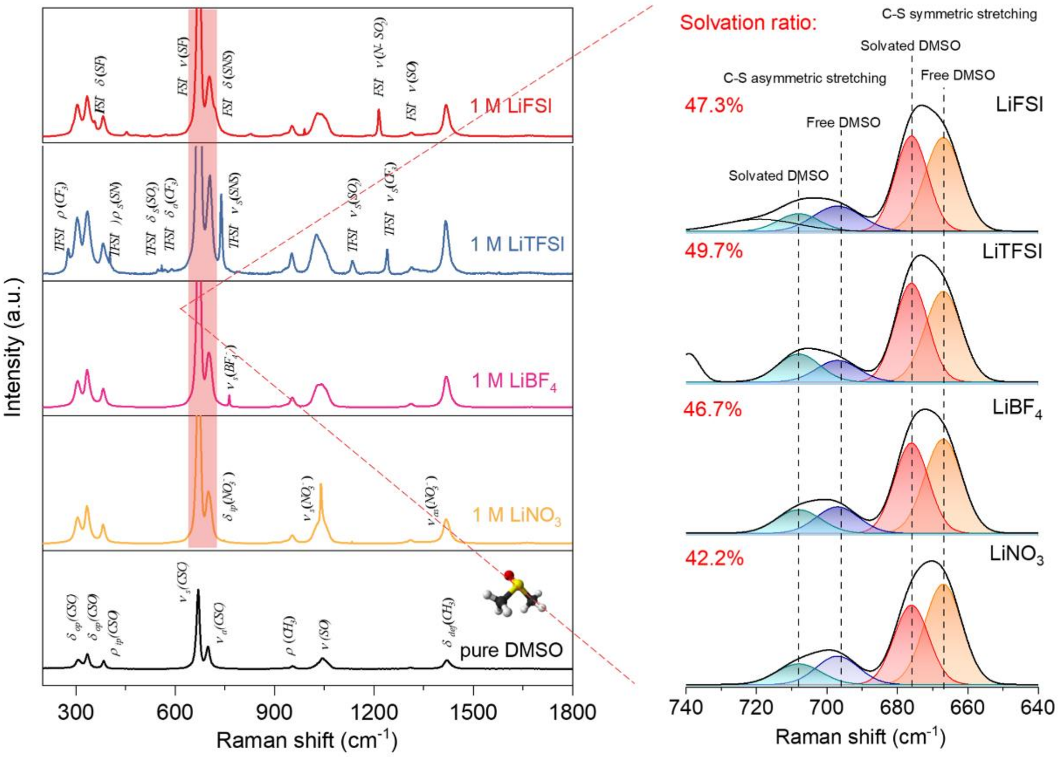

Supplementary Fig. 31. Raman spectra of pure DMSO and 1 M concentrations of different lithium salts in DMSO solvent in the range of 200-1800 cm^{-1} (left) and enlargement for the range of 640-740 cm^{-1} (right).[8-11] The peaks located at 667 and 697 cm^{-1} are from the vibrations of C-S symmetric and asymmetric stretching modes from free DMSO, respectively; the two solvated peaks at 676 and 708 cm^{-1} are assigned to C-S symmetric and asymmetric stretching modes of DMSO molecules that were solvated with Li^+ ions. The solvation ratios of these various electrolytes are marked by red numbers.

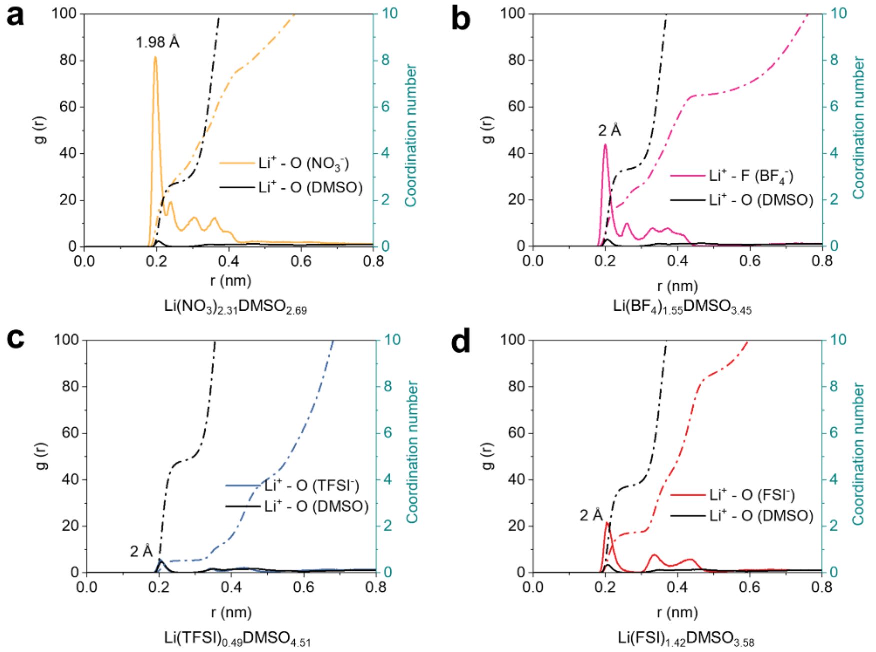

Supplementary Fig. 32. The radial distribution functions (RDFs) of Li-DMSO/anions and their coordination numbers in 1 M (a) $LiNO_3$, (b) $LiBF_4$, (c) LiTFSI, and (d) LiFSI electrolytes. The dashed-dotted lines express the coordination numbers.

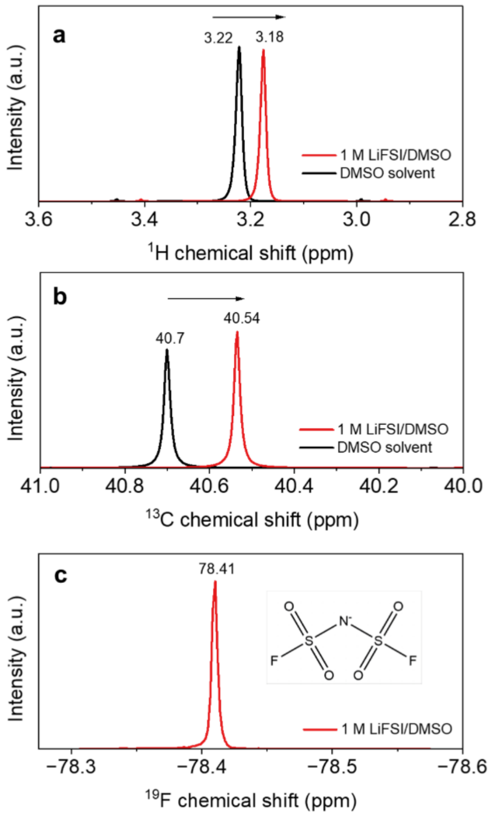

Supplementary Fig. 33. NMR analysis for solutions: (a) ^{1}H NMR, and (b) ^{13}C NMR spectra of pure DMSO solvent and 1 M LiFSI/DMSO electrolytes. (c) ^{19}F NMR spectrum of and 1 M LiFSI/DMSO electrolytes.

Note: An upfield (more negative) shift in ^{1}H and ^{13}C NMR spectra, respectively, could indicate Li-solvent binding interaction, confirming the existence of Li^{+} solvation shells. In addition, the appearance of distinct ^{19}F NMR peaks were observed and attributed to the Li^{+}-FSI^{-} coordination.

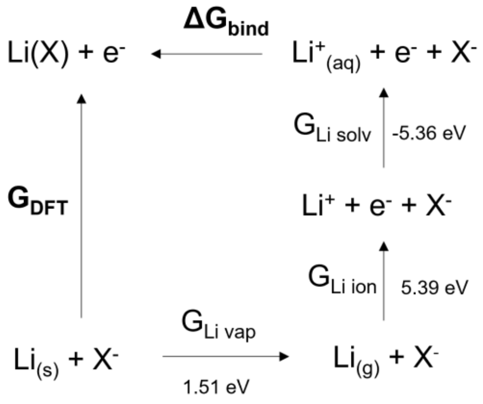

Supplementary Fig. 34. Thermodynamic cycle of the charge-transfer reactions involving Li^+ and other electrolyte components: an anion (X) and DMSO, used to calculate the binding energy (ΔG_{bind}) between Li^+ and associated species. The energies for Li vaporisation, Li ionisation, and Li solvation in organic electrolytes were used as in the previous model.[12,13] A Li solvation energy in DMSO of -5.36 eV was used.[14]

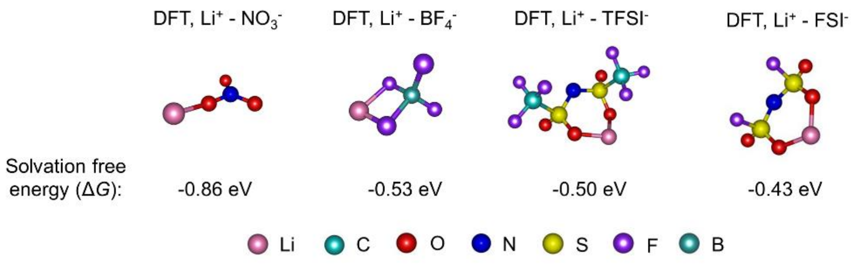

Supplementary Fig. 35. Binding free energy between Li^+ ion and different anions, as calculated by DFT. Colour scheme of molecules: Li, pink; C, light blue; H, white; O, red; N, navy; S, yellow; F, purple; and B, blue.

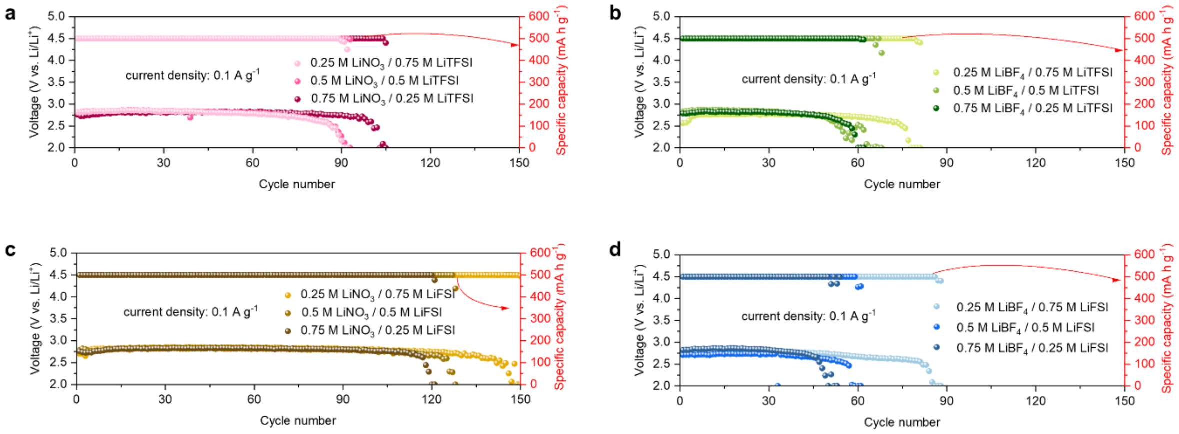

Supplementary Fig. 36. Long-term cycling performance of Li-CO_2 cells in various dual-salt electrolytes at a current density of 0.1 A g^{-1} with a cut-off specific capacity of 500 mA h g^{-1} (in the cut-off voltage from 2 V to 5 V) in (a) $LiNO_3$/LiTFSI electrolytes, (b) $LiBF_4$/LiTFSI electrolytes, (c) $LiNO_3$/LiFSI electrolytes, and (d) $LiBF_4$/LiFSI electrolytes.

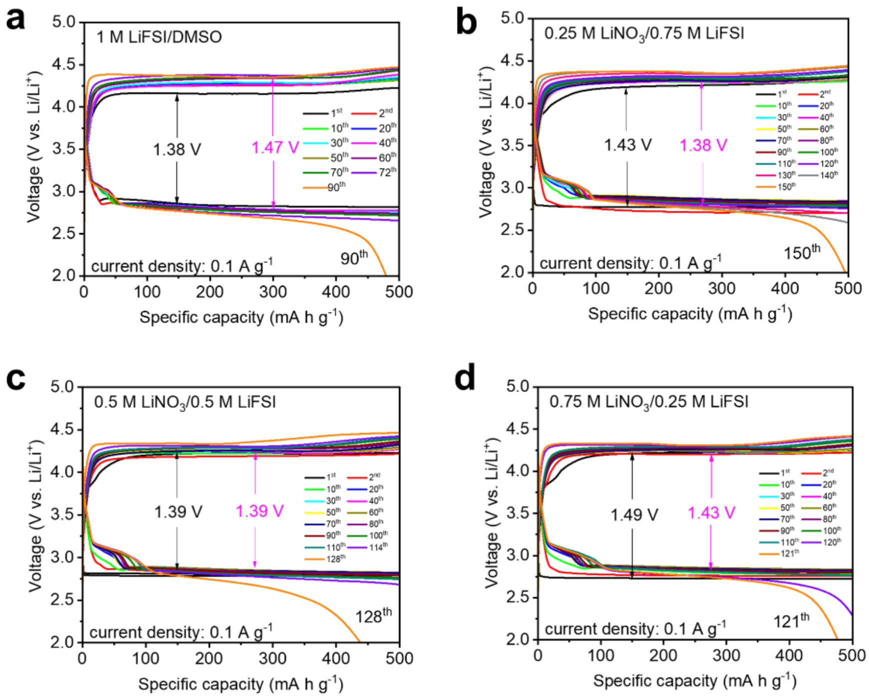

Supplementary Fig. 37. Cycling performance of Li-CO_2 batteries at a current density of 0.1 A g^{-1} with a cut-off specific capacity of 500 mA h g^{-1} (in the cut-off voltage from 2 V to 5 V) in (a) 1 M LiFSI, (b) 0.25 M $LiNO_3$/0.75 M LiFSI, (c) 0.5 M $LiNO_3$/0.5 M LiFSI, and (d) 0.75 M $LiNO_3$/0.25 M LiFSI electrolytes.

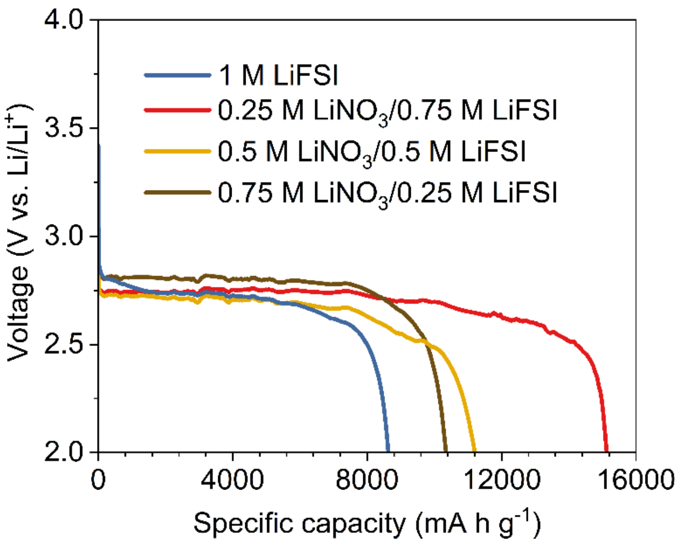

Supplementary Fig. 38. Full-discharge curves (at a current density of 0.1 A g^{-1} with low cut-off voltage of 2 V) of Li-CO_2 batteries in 1 M single-salt LiFSI and dual-salt electrolytes.

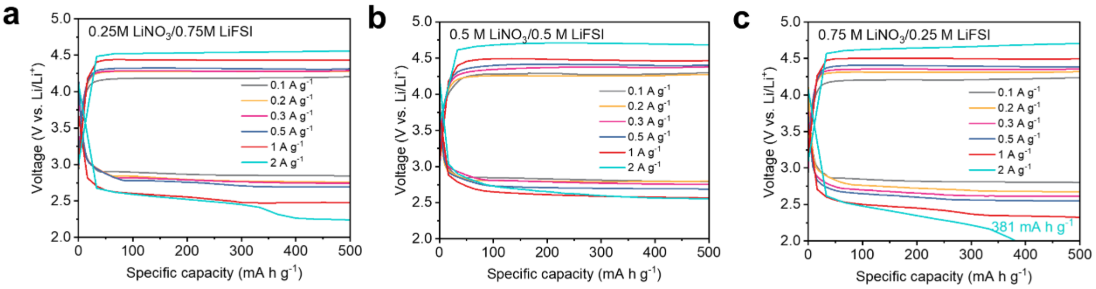

Supplementary Fig. 39. Rate performance of Li-CO_2 batteries at various current densities with a cut-off specific capacity of 500 mA h g^{-1} (in the cut-off voltage from 2 V to 5 V) in (a) 0.25 M $LiNO_3$/0.75 M LiFSI, (b) 0.5 M $LiNO_3$/0.5 M LiFSI, and (c) 0.75 M $LiNO_3$/0.25 M LiFSI electrolytes.

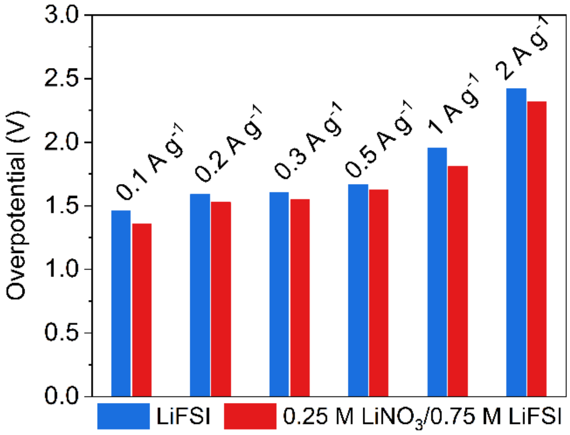

Supplementary Fig. 40. Overpotential comparison of the 1 M single-salt LiFSI electrolyte and the 0.25 M $LiNO_3$/0.75 M LiFSI electrolyte at various current densities.

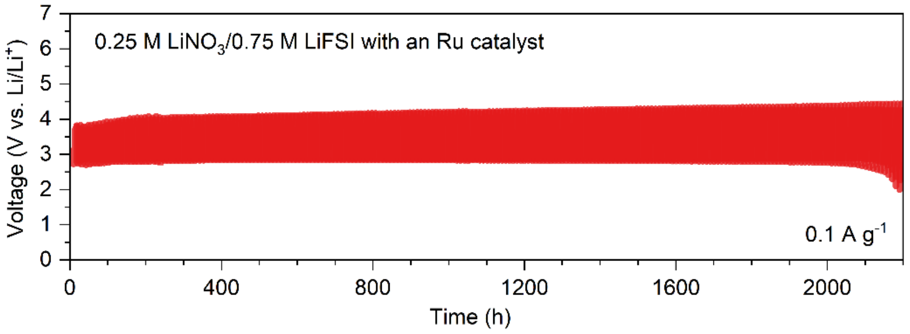

Supplementary Fig. 41. Long-term cycling performance of the Li-CO_2 cell using the 0.25 M $LiNO_3$/0.75 M LiFSI electrolyte with an Ru catalyst at 0.1 A g^{-1} in the cut-off voltage from 2 V to 5 V.

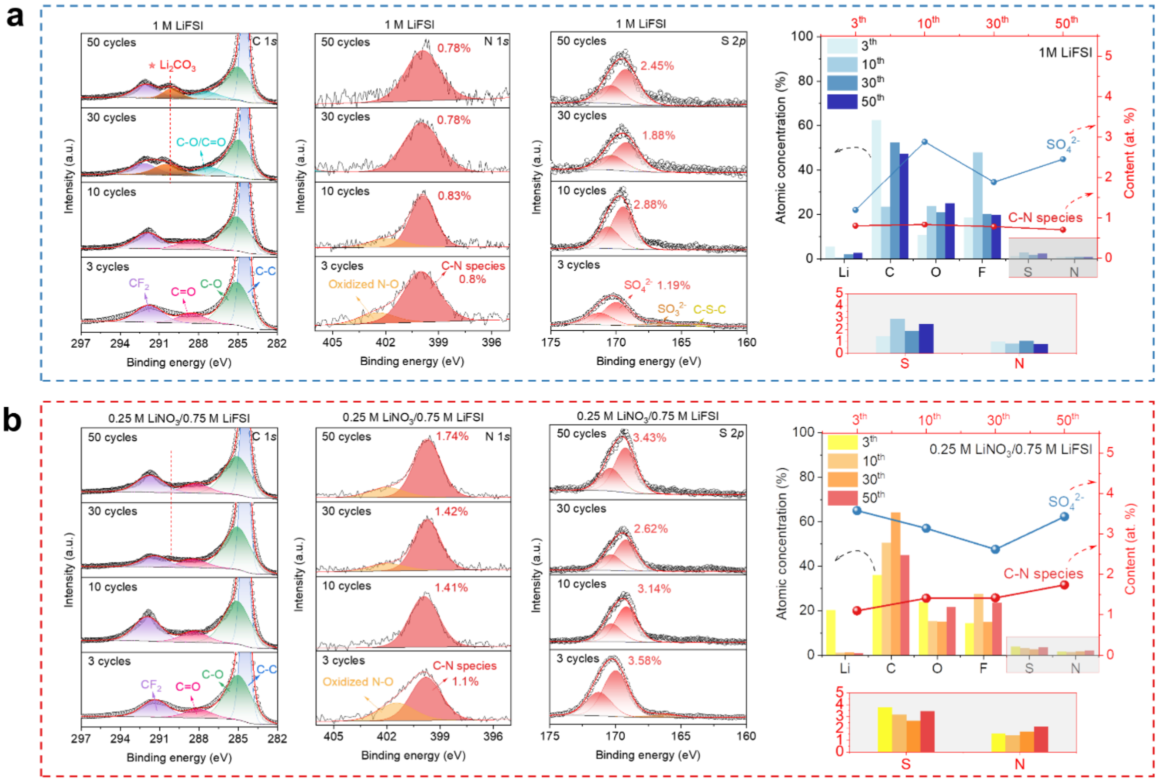

Supplementary Fig. 42. Characterization of SEI composition on the cathodes cycled in Li-CO_2 batteries. C 1*s*, N 1*s*, and S 2*p* XPS spectra, and Comparison of C-N species and SO_4^{2-} content for the cathodes after 3, 10, 30, and 50 cycles in the cells using (a) single-salt LiFSI and (b) 0.25 M $LiNO_3$/0.75 M LiFSI electrolytes (atomic concentrations of S and N elements are shown in the insets).

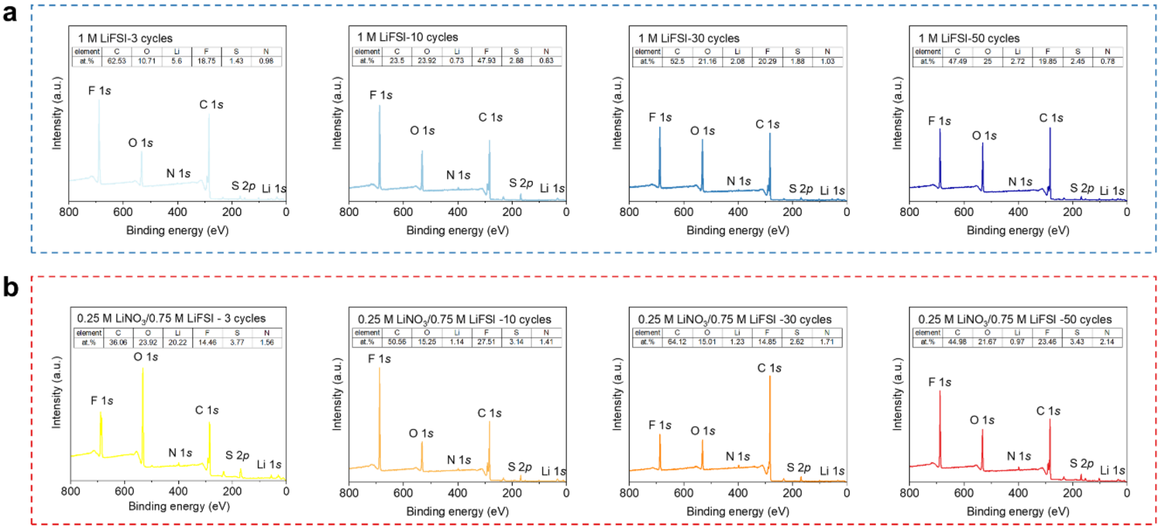

Supplementary Fig. 43. XPS survey spectra of the cathodes after 3, 10, 30, and 50 cycles in the cells using (a) single-salt LiFSI and (b) 0.25 M $LiNO_3$/0.75 M LiFSI electrolytes.

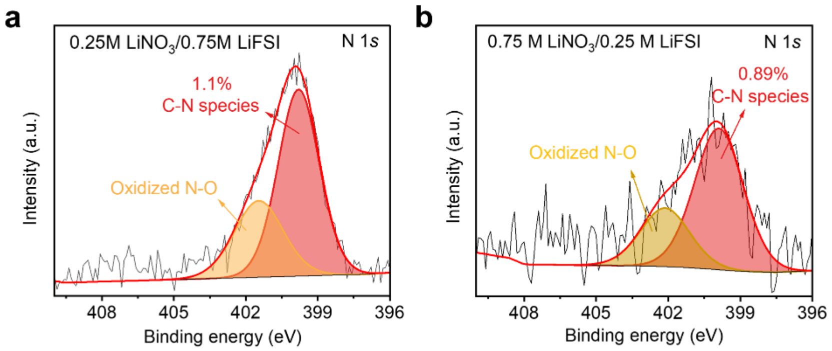

Supplementary Fig. 44. N 1*s* XPS spectra of the cathodes after 3 cycles in the cells using (a) 0.25 M $LiNO_3$/0.75 M LiFSI and (b) 0.75 M $LiNO_3$/0.25 M LiFSI electrolytes.

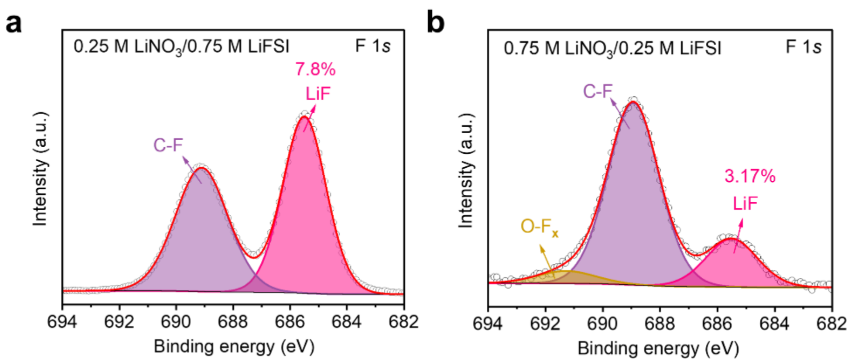

Supplementary Fig. 45. F 1*s* XPS spectra of the cathodes after 3 cycles in the cells using (a) 0.25 M $LiNO_3$/0.75 M LiFSI and (b) 0.75 M $LiNO_3$/0.25 M LiFSI electrolytes.

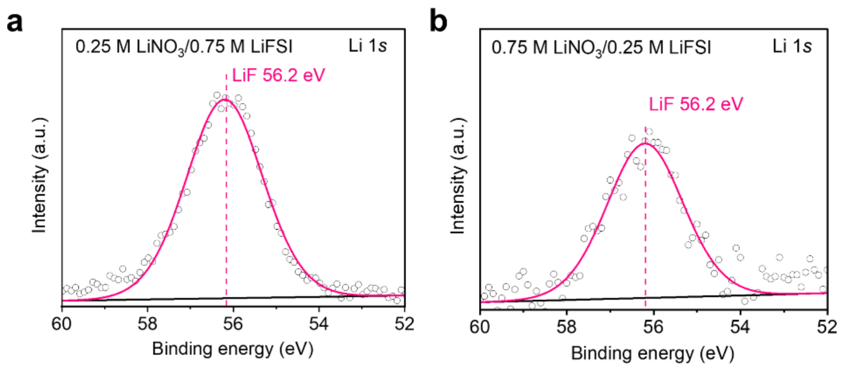

Supplementary Fig. 46. Li 1*s* XPS spectra of the cathodes after 3 cycles in the cells using (a) 0.25 M $LiNO_3$/0.75 M LiFSI and (b) 0.75 M $LiNO_3$/0.25 M LiFSI electrolytes.

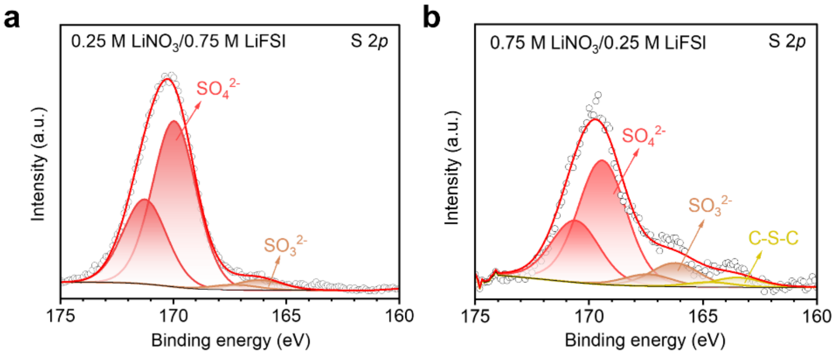

Supplementary Fig. 47. S 2*p* XPS spectra of the cathodes after 3 cycles in the cells using (a) 0.25 M $LiNO_3$/0.75 M LiFSI and (b) 0.75 M $LiNO_3$/0.25 M LiFSI electrolytes.

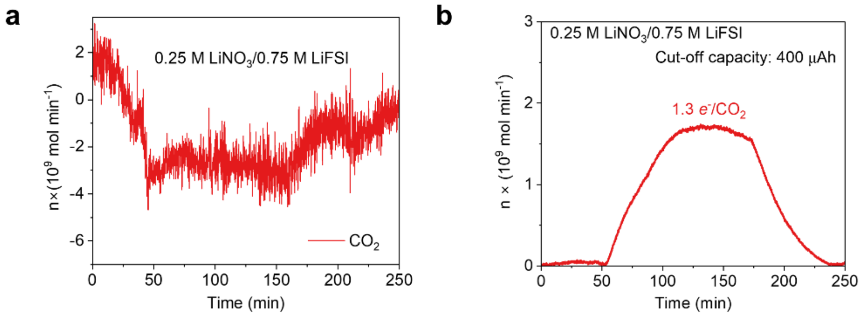

Supplementary Fig. 48. *In-situ* DEMS analysis of Li-CO_2 cells in the 0.25 M $LiNO_3$/0.75 M LiFSI electrolyte in the first (a) discharge and (b) recharge processes tested at 200 µA within the capacity of 400 µAh.

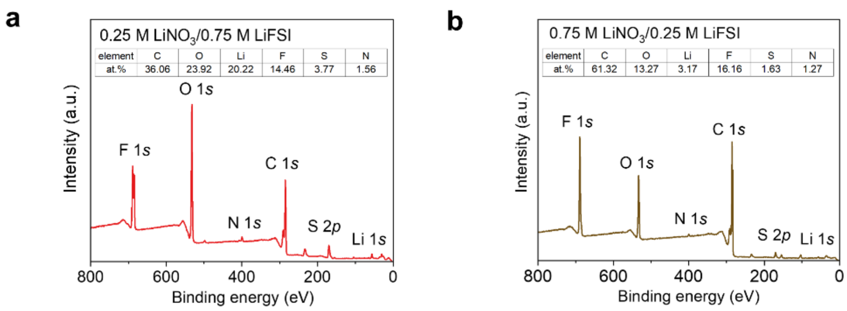

Supplementary Fig. 49. XPS survey spectra of the cathodes after 3 cycles in the cells using (a) 0.25 M $LiNO_3$/0.75 M LiFSI and (b) 0.75 M $LiNO_3$/0.25 M LiFSI electrolytes.

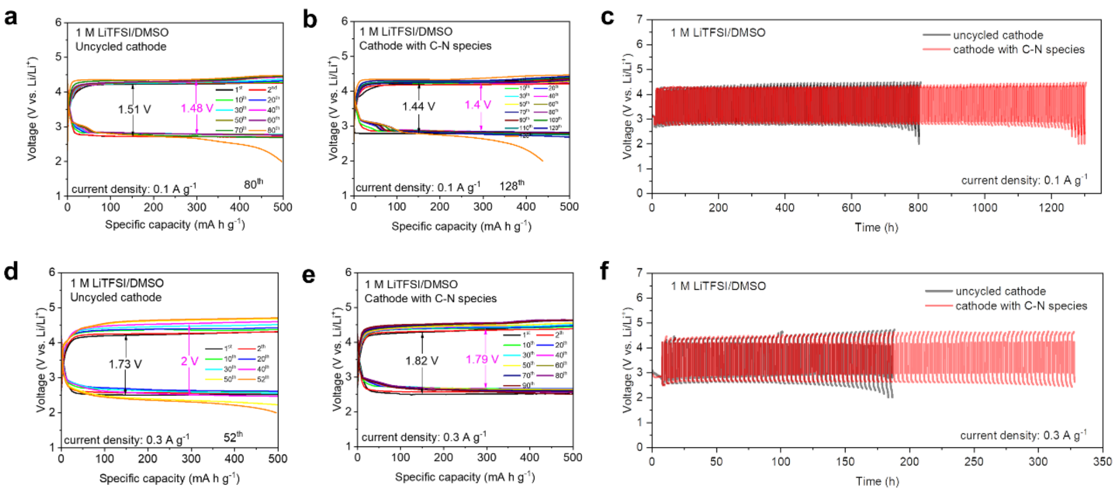

Supplementary Fig. 50. Cycling performance of Li-CO_2 batteries in conventional 1 M LiTFSI/DMSO electrolytes (a) using uncycled cathodes, (b) using cathodes with C-N species, and (c) corresponding long-term voltage-time profiles at 0.1 A g^{-1} in the cut-off voltage from 2 V to 5 V; (d) using uncycled cathodes, (e) using cathodes with C-N species, and (f) corresponding long-term voltage-time profiles at 0.3 A g^{-1}.

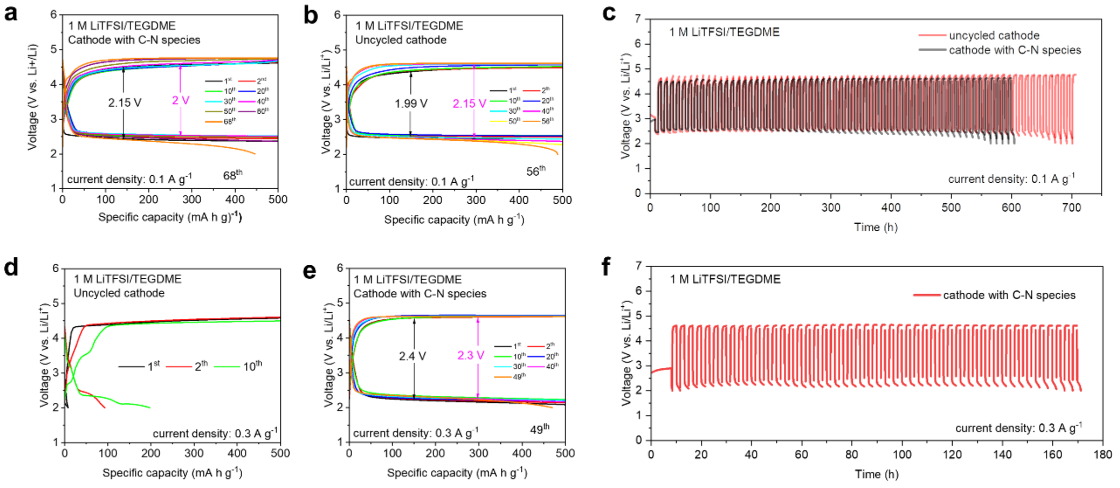

Supplementary Fig. 51. Cycling performance of Li-CO_2 batteries in conventional 1 M LiTFSI/TEGDME electrolytes (a) using uncycled cathodes, (b) cathodes with C-N species, and (c) corresponding long-term voltage-time profiles at 0.1 A g^{-1} in the cut-off voltage from 2 V to 5 V; (d) using uncycled cathodes, (e) using cathodes with C-N species, and (f) corresponding long-term voltage-time profiles at 0.3 A g^{-1}.

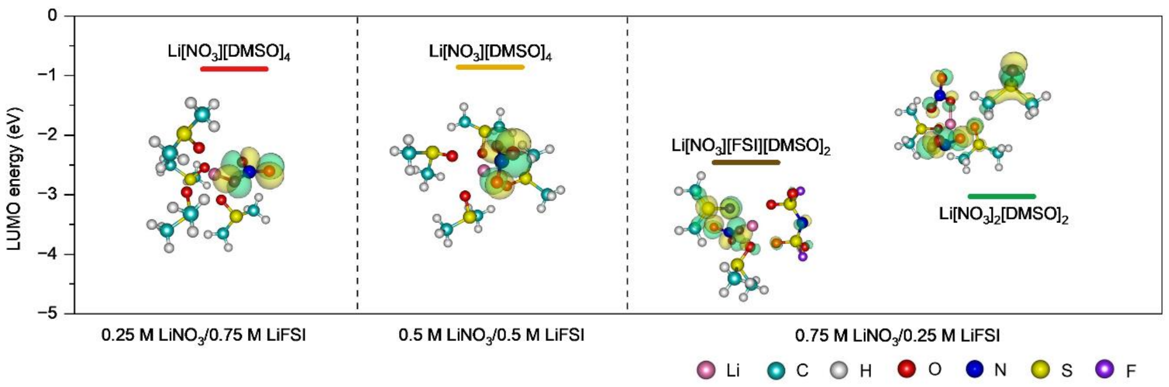

Supplementary Fig. 52. Other probable structures of Li^+ solvation clusters and the corresponding lowest unoccupied molecular orbital (LUMO) energy values in dual-salt electrolytes. Colour scheme of molecules: Li, pink; C, light blue; H, white; O, red; N, navy; S, yellow; F, purple; and B, blue. The yellow and blue zones represent electron loss and gain, respectively.

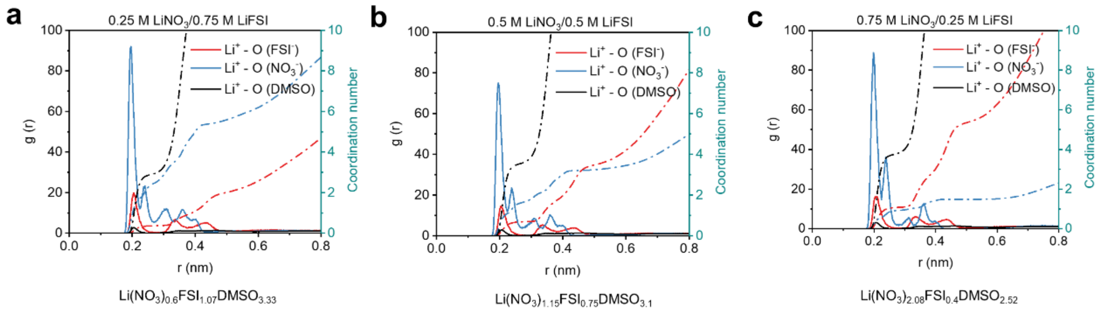

Supplementary Fig. 53. The RDFs of Li-DMSO/anions and their coordination numbers in (a) 0.25 M $LiNO_3$/0.75 M LiFSI, (b) 0.5 M $LiNO_3$/0.5 M LiFSI, and (c) 0.75 M $LiNO_3$/0.25 M LiFSI electrolytes. The dashed-dotted lines represent the coordination numbers.

Supplementary Tables

Supplementary Table 1. The adsorption energies between CO_2, $*CO_2^{2-}$, and Li_2CO_3 and atomic cathode surface layer of graphene, C-S, C-O, C-N, and C-F.

Adsorption Energy (eV)	Graphene	C-O	C-S	C-N	C-F
CO_2	0.21	0.21	0.21	0.10	0.20
$*CO_2^{2-}$	0.27	-0.06	0.33	-1.04	-2.11
Li_2CO_3	-0.38	-0.53	-0.34	-1.44	-1.43

Supplementary Table 2. Interaction models between cations and anions, and corresponding binding wavenumbers of vibrations of various anions.

Anions	Free/cm^{-1}	SSIP/cm^{-1}	CIP/cm^{-1}
NO_3^-[15]	1039	1045	1051
BF_4^-[16]	760	763	768
$TFSI^-$[17]	736-738	740-742	744-746
FSI^-[18,19]	719	722	731

Supplementary Table 3. Fitting results for Raman spectra.

Samples	Content in solvent (%)		Content of anions (%)		
	Free	Solvation	Free	SSIP	CIP
1M $LiNO_3$	57.75	42.25	42.5	0	52.5
1M $LiBF_4$	53.25	46.75	0	87.6	12.4
1M LiTFSI	50.31	49.96	35.1	51.2	13.7
1M LiFSI	52.7	47.3	0	55.35	44.65

Supplementary Table 4. The percentages of different Li^+ solvation clusters in 1 M $LiNO_3$, $LiBF_4$, LiTFSI, and LiFSI electrolytes, calculated by MD.

Li^+ solvation cluster	NO_3^-	BF_4^-	$TFSI^-$	FSI^-
Solvent-surrounded Li^+	8%	23%	56%	14%
Li^+-single anion pair	37%	33%	38%	50%
Li^+-multiple anion cluster	55%	43%	6%	36%

Supplementary Table 5. Comparison of the physical/chemical properties and prices of different lithium salts. DN: donor number.

Salts	Molecular structure	Radius (Å)[20,21]	DN (kcal/mol)[22-24]	Price (AU$)
$LiNO_3$	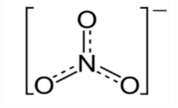	2.33	21.1	66.3/100g
LiFSI	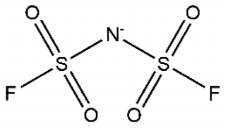	3.27	9.5	210/100g
$LiBF_4$	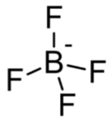	2.4	6.03	326/50g
LiTFSI	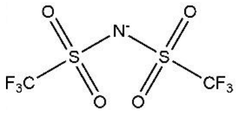	3.53	5.4	579/100g

Supplementary Table 6. Comparison of the cycling performance of Li-CO_2 cells using uncycled cathodes and cathodes with C-N species.

Conventional Electrolytes	Cathodes	Cycle life<br>0.1 A g^{-1}	Cycle life<br>0.3 A g^{-1}
1 M LiTFSI/DMSO	Uncycled cathodes	80	82
	Cathodes with C-N species	128	94
1 M LiTFSI/TEGDME	Uncycled cathodes	56	-
	Cathodes with C-N species	68	49

Supplementary Table 7. The percentages of different Li^+ solvation clusters in dual-salt electrolytes calculated by MD.

Li^+ solvation cluster	NO_3^-:FSI^- = 1:3	NO_3^-:FSI^- = 1:1	NO_3^-:FSI^- = 3:1
Solvent-surrounded Li^+	14%	12%	7%
Li^+-NO_3^--FSI^- cluster	14%	23%	27%
Li^+-NO_3^-	35%	60%	85%
Li^+-single NO_3^- pair	21%	27%	30%
Li^+-NO_3^- cluster (≥2)	0	10%	28%
Li^+-FSI^-	64%	51%	36%
Li^+-single FSI^- pair	36%	25%	7%
Li^+-FSI^- cluster (≥2)	14%	3%	2%

Supplementary References

1. Tetsuka, H. et al. Molecularly designed, nitrogen-functionalized graphene quantum dots for optoelectronic devices. *Adv. Mater.* **28**, 4632-4638 (2016).

2. Permatasari, F.A. et al. Design of pyrrolic-N-rich carbon dots with absorption in the first near-infrared window for photothermal therapy. *ACS Appl. Nano Mater.* **1**, 2368-2375 (2018).

3. Yang, J. et al. Adsorption-catalysis synergy within pyrrolic-N-rich carbon nanosheets: Propelling electrochemical kinetics and shielding polysulfides for lithium-sulfur batteries. *Chem. Eng. J.* **476**, 146532 (2023).

4. Li, Y. et al. Highly surface-wrinkled and N-doped CNTs anchored on metal wire: A novel fiber-shaped cathode toward high-performance flexible Li–CO_2 batteries. *Adv. Funct. Mater.* **29**, 1808117 (2019).

5. Zhang, J. et al. Rechargeable Li-CO_2 Batteries with Graphdiyne as Efficient Metal-Free Cathode Catalysts. *Adv. Funct. Mater.* **31**, 2101423 (2021).

6. Qiao, Y. et al. 3D-Printed Graphene Oxide Framework with Thermal Shock Synthesized Nanoparticles for Li-CO_2 Batteries. *Adv. Funct. Mater.* **28**, 1805899 (2018).

7. Hwang, J.Y., Park, S.J., Yoon, C.S. & Sun, Y.K. Customizing a Li–metal battery that survives practical operating conditions for electric vehicle applications. *Energy Environ. Sci.* **12**, 2174-2184 (2019).

8. Miller, R.E., Getty, R.R., Treuil, K.L. & Leroi, G.E. Spectrum of Crystalline Lithium Nitrate. *J. Chem. Phys.* **51**, 1385-1389 (1969).

9. Allen, J.L., Han, S.D., Boyle, P.D. & Henderson, W.A. Crystal structure and physical properties of lithium difluoro(oxalato)borate (LiDFOB or $LiBF_2O_x$). *J. Power Sources.* **196**, 9737-9742 (2011).

10. Wang, L., Uosaki, K. & Noguchi, H., Effect of Electrolyte Concentration on the Solvation Structure of Gold/LITFSI–DMSO Solution Interface. *J. Phys. Chem. C.* **124**, 12381-12389 (2020).

11. Li, L. et al. Transport and electrochemical properties and spectral features of non-aqueous electrolytes containing LiFSI in linear carbonate solvents. *J. Electrochem. Soc.* **158**, 74 (2010).

12. Yuwono, J.A. et al. Atomistic insights into lithium storage mechanisms in anatase, rutile, and amorphous TiO_2 electrodes. *ACS Appl. Mater. Interfaces.* **13**, 1791-1806 (2021).

13. Leung, K. & Tenney, C.M. Toward first principles prediction of voltage dependences of electrolyte/electrolyte interfacial processes in lithium ion batteries. *J. Phys. Chem. C.* **117**, 24224-24235 (2013).

14. Carvalho, N.F. & Pliego, J.R., Cluster-continuum quasichemical theory calculation of the lithium ion solvation in water, acetonitrile and dimethyl sulfoxide: an absolute single-ion solvation free energy scale. *Phys Chem Chem Phys.* **17**, 26745-26755 (2015).

15. Wang, X. et al. Hybrid Electrolyte with Dual-Anion-Aggregated Solvation Sheath for Stabilizing High-Voltage Lithium-Metal Batteries. *Adv. Mater.* **33**, 2007945 (2021).

16. Kirillov, S.A., Gafurov, M.M., Gorobets, M.I. & Ataev, M.B. Raman study of ion pairing in solutions of lithium salts in dimethyl sulfoxide, propylene carbonate and dimethyl carbonate. *J. Mol. Liq.* **199**, 167-174 (2014).

17. Suo, L., Zheng, F., Hu, Y.S. & Chen, L. FT-Raman spectroscopy study of solvent-in-salt electrolytes. *Chinese Phys. B*. **25**, 016101 (2016).

18. Yao, Y. X. et al. Regulating Interfacial Chemistry in Lithium-Ion Batteries by a Weakly Solvating Electrolyte. *Angew. Chem. Int. Ed*. **60**, 4090-4097 (2021).

19. Chang, Z. h. et al. Effect of Dual-Salt Concentrated Electrolytes on the Electrochemical Performance of Silicon Nanoparticles. *ChemElectroChem* **7**, 1135-1141 (2020).

20. Han, K. S. et al. Effects of Anion Mobility on Electrochemical Behaviors of Lithium–Sulfur Batteries. *Chem. Mater*. **29**, 9023-9029 (2017).

21. Johansson, P. Electronic structure calculations on lithium battery electrolyte salts. *Phys. Chem. Chem. Phys*. **9**, 1493-1498 (2007).

22. Chu, H. et al. Achieving three-dimensional lithium sulfide growth in lithium-sulfur batteries using high-donor-number anions. *Nat. Commun*. **10**, 188 (2019).

23. Kaiser, M. R. et al. Structure-Property Relationships of Organic Electrolytes and Their Effects on Li/S Battery Performance. *Adv. Mater*. **29**, 1700449 (2017).

24. Wang, Z. et al. Non-Flammable Ester Electrolyte with Boosted Stability Against Li for High-Performance Li metal Batteries. *Angew. Chem. Int. Ed*. **61**, 202206682 (2022).
